# Efficacy and safety of immune checkpoint inhibitors for advanced squamous non-small cell lung cancer: a systematic review and network meta-analysis

**DOI:** 10.3389/fimmu.2025.1635757

**Published:** 2025-09-16

**Authors:** Na Liu, Baowanze Zhang, Jiali He, Su Li

**Affiliations:** Department of Pharmacy, Cancer Hospital of China Medical University, Liaoning Cancer Hospital and Institute, Shenyang, Liaoning, China

**Keywords:** advanced squamous non-small cell lung cancer, immunotherapy, efficacy, safety, network meta-analysis

## Abstract

**Objective:**

Significant efficacy heterogeneity exists between first- and second-line immunotherapy regimens for advanced squamous non-small cell lung cancer (SqNSCLC), but most regimens lack directly comparable clinical trial evidence, resulting in unclear prioritization. This analysis identifies optimal treatment strategies by evaluating differences in efficacy across immune checkpoint inhibitors (ICIs).

**Methods:**

We search through comprehensive databases, including PubMed, Embase, the Cochrane Library and the Clinical Trials Database. Traditional meta-analysis was done using Stata 15.0, while Bayesian-framework network meta-analysis was implemented with R’s GEMTC package via Markov chain Monte Carlo simulation. Subgroup analyses were performed for different PD-L1 expression levels, number of treatments, ethnic groups, and smoking history.

**Results:**

We included 25 randomized controlled trials. Immune-related therapy can provide significant benefit relative to chemotherapy alone in advanced SqNSCLC. Compared with chemotherapy, except for ipilimumab+chemo [HR = 0.92,95%CI: (0.59-1.40)], atezolizumab+chemo [HR = 0.88, 95%CI: (0.56-1.40)], and durvalumab+chemo [HR = 0.84, 95% CI: (0.52-1.40)], durvalumab+ tremelimumab+chemo [HR = 0. 88, 95% CI: (0.54-1.40)], which significantly improved overall survival(OS). Cemiplimab [HR = 0.48, 95% CI: (0.34-0.67)] showed the best OS benefit. Compared with chemotherapy, all immunotherapies significantly improved progression-free survival (PFS) except for ipilimumab+chemo [HR = 0.87, 95% CI: (0.75-1.00)]. Sugemalimab+chemo provided the best survival benefit [HR = 0.34, 95% CI: (0.24-0.48)]. For PD-L1≥50% tumors, penpulimab showed excellent OS and PFS; for PD-L1 1-49% tumors, pembrolizumab+chemo and camrelizumab+chemo achieved the best OS and PFS, respectively; for PD-L1≥1% tumors, the tislelizumab+chemo and camrelizumab+chemo showed the best OS and PFS results, while for tumors with PD-L1 <1%, both nivolumab and serplulimab+chemo provided significant survival benefit. In Asian patients, patients treated with pembrolizumab or pembrolizumab + chemotherapy had favorable OS and PFS benefits. In non-Asian patients, there was also favorable OS and PFS benefit with cemiplimab. For former/current smokers, pembrolizumab+chemotherapy and camrelizumab+chemotherapy had significant OS and PFS benefit, but most immunotherapies did not improve OS and PFS in never smokers. Camrelizumab+chemo [OR = 3.5, 95% CI: (2.3-5.3)] had the best overall response rate (ORR) benefit. Ipilimumab+chemo had the highest incidence of adverse events (AEs) [OR = 2.0, 95% CI:(1.5-2.7)].

**Systematic review registration:**

https://www.crd.york.ac.uk/prospero/, identifier CRD420251027447.

## 1 Introduction

Lung cancer remains the leading contributor to global cancer-related deaths (1). NSCLC constitutes over 80% of incident cases, of which squamous histology accounts for nearly one-third (2). The lack of typical clinical manifestations in the early stages of squamous lung cancer results in about 80% of cases being detected at advanced stages, when surgical treatment is no longer optimal. Most squamous lung cancers do not have a clear driver gene and have a low probability of being suitable for targeted drug therapy (3). Therefore, chemotherapy retains its fundamental position in the standard care regimen. For SqNSCLC, platinum-based dual-agent regimens remain the cornerstone of frontline therapy, integrating platinum compounds with mechanistically distinct cytotoxic agents. Second-line therapeutic strategies typically employ docetaxel monotherapy. However, the survival outcome benefit remains poor due to the rapid emergence of resistance and transient benefit of treatment. Conventional platinum-based doublet chemotherapy demonstrates suboptimal PFS and OS (4–7). Therefore, the above situation has forced doctors and scientists to seek better treatment options.

Immunotherapy has transformed therapeutic paradigms across solid malignancies. PD-1/PD-L1 antagonists counteract tumor-driven immunosuppression by blocking molecular interactions between cancer cells and T lymphocytes, reinstating antitumor immune responses (8). CTLA-4 blockers inhibit early-phase immune regulation by preventing receptor-ligand engagement during antigen presentation, simultaneously amplifying effector T-cell activity and diminishing immunosuppressive regulatory T-cell functions (9). Chemotherapy combined with immunotherapy regimens such as nivolumab, pembrolizumab, and atezolizumab have been established as preferred first-line therapy for SqNSCLC. In addition to monotherapy, combination therapeutic strategies with ICIs have been adopted clinically because of their potential for synergistic antitumor effects and improved clinical outcomes (10). Emerging clinical evidence from recent trials has further validated the efficacy of multiple ICI-based combination therapies, including dual immunotherapy (e.g., PD-1/CTLA-4 inhibitors) and chemo-immunotherapy regimens (11).

To date, the lack of direct or indirect comparisons between these agents has left physicians in a difficult position when making clinical decisions about which treatment regimen to choose. Furthermore, therapeutic approaches demonstrate marked heterogeneity in safety profiles, with immune-mediated adverse events presenting significant risks for this patient cohort, necessitating prompt initiation of dedicated research initiatives. We analyzed SqNSCLC immunotherapy regimens comprehensively, assessed their treatment effects, and provided guidance for clinical decision-making.

## 2 Methods

### 2.1 Literature search

The literature search encompassed PubMed, The Cochrane Library and Embase databases. Review articles and references of the included studies and trial registry databases (clinicaltrials.gov) were also searched for additional relevant information. Literature reviews covered studies up to January 2025, with no restriction on their original publication date. **Supplementary Table S1** outlines the PubMed-based search methodology, designed in alignment with PRISMA standards for systematic reviews and meta-analyses. The study protocol was prospectively registered in PROSPERO prior to implementation (CRD420251027447).

### 2.2 Inclusion and exclusion criteria

Inclusion criteria:

1. Phases II and III of the randomized, controlled clinical trials;
2. Participant type: patients with stage IIIB/IV SqNSCLC without a driver gene or an enrollment population that includes patients with advanced SqNSCLC.
3. One or more ICI-based treatment regimens were used in the experimental group and chemotherapy in the control group.
4. The primary clinical outcomes assessed in the Phase II/III trial included OS, PFS, ORR and grade ≥3 AEs.

Exclusion criteria:

1. Duplicate published literature;
2. Research on non-ICI treatment options, such as radiation therapy, surgery, and targeted therapy.
3. Case studies, basic laboratory experiments, conference abstracts, animal experiments, and other studies.

### 2.3 Data extraction

Two authors collected data independently, and differences were resolved by discussion and negotiation or by recourse to the corresponding author. For each clinical trial, the following details were systematically recorded: name of the primary study, year of publication, intervention and comparison groups, type of study, treatment regimen and corresponding number of people, inclusion and exclusion criteria, median age of study participants, sex ratio, hazard ratios (HRs) with 95% CIs were analyzed for median OS and PFS, while odds ratios (ORs) with 95% CIs assessed ORR and grade ≥3 adverse events.

### 2.4 Quality assessment of included studies

All 25 trials were assessed using the Cochrane Risk of Bias Assessment Tool (RevMan5.4) across seven dimensions, with the researchers’ assessment of risk of bias categorized into three levels: low risk, high risk and unclear.

### 2.5 Credibility assessment

We used the Confidence in Network Meta-Analysis (CINeMA) web tool to assess the confidence of each comparison. Following recommended guidelines, we assessed six factors for each comparison: within-study bias, reporting bias, indirectness, imprecision, heterogeneity, and inconsistency. Consistent with the GRADE framework, the quality of evidence for each comparison was categorized as high, moderate, low, and very low.

### 2.6 Data analysis

We used the R language (version 4.4.3) in a Bayesian framework based on the JAGS package and GEMTC package 25 randomized controlled trials were analyzed for NMA. We used consistent and non-consistent models for DIC comparisons; if the DICs were similar, the consistent model was chosen, and if the DICs were too different, the non-consistent model was chosen. At the same time, the model was built using Markov chain Monte Carlo method using R language in a Bayesian framework. In addition, the efficacy of ICIs was ranked using the Surface Under the Cumulative Ranking Probability Curve (SUCRA), which has a value between 0 and 1, with larger values indicating better efficacy. We used Stata MP 15.1 for general meta-analysis. Study heterogeneity was evaluated through p-value and I²statistics, with meta-analysis models selected based on predefined thresholds: fixed-effects for homogeneity (P > 0.10, I²< 50%) or random-effects for heterogeneity (P ≤ 0.10, I² ≥ 50%). Further subgroup analyses were performed based on treatment regimen, PD-L1 expression level, patient ethnicity, and smoking history.

## 3 Results

### 3.1 Eligible studies and characteristics

**Figure 1:**
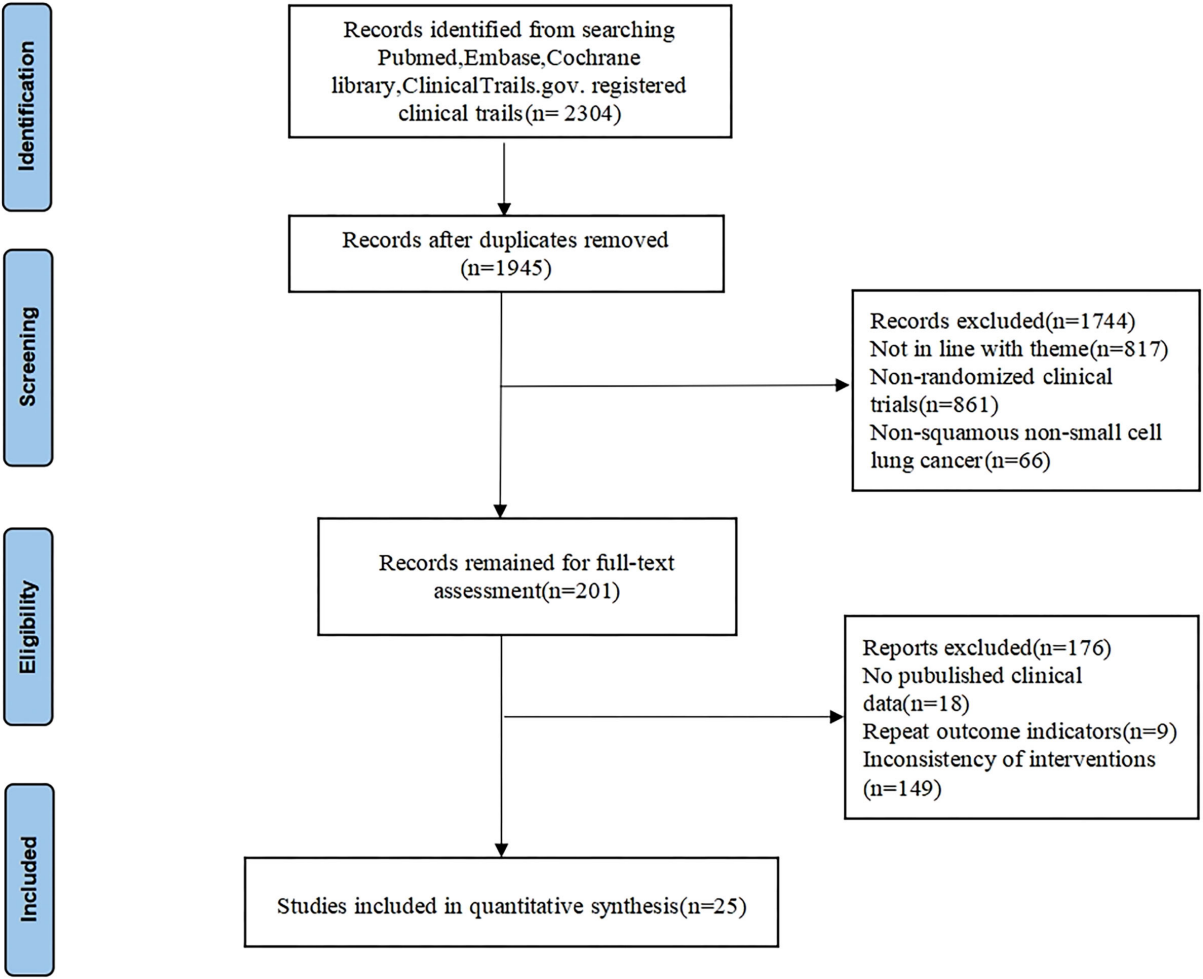
Flow diagram of the study selection process.

We conducted a comprehensive search in various databases and found 2304 relevant articles. Following duplicate removal, a total of 1945 articles were retained, 1744 irrelevant articles were eliminated by reading the titles and abstracts, 176 articles were eliminated for further reading due to unavailability of the original article, incomplete data, duplication of clinical data in the study, and non-compliance of the interventions, etc. Ultimately, 25 studies (12–36) were included in the analysis, including 13903 patients. The detailed screening process is illustrated in **Figure 1**. The CheckMate 017, Study 104, CameL-sq, ORIENT-12, ASTRUM-004, AK105-302, and ORIENT-3 RCTs restricted enrollment to SqNSCLC patients, whereas other RCTs permitted both squamous and non-squamous histologies yet reported SqNSCLC subgroup analyses separately. The baseline characteristics and results for each trial are shown in **Table 1**.

**Table 1:** Characteristics of included studies.

Study	Year	Phase	Stage	Sample size	Median age	Male/ Female	Experimental arm	Control arm
IMpower 110 (NCT02409342) (12)	2020	Phase III,Open Label	IV	285/287	63.1/63.8	396/176	Atezolizumab 1250mg/m^2^ Q3W	Chemotherapy(Carboplatin AUC 5 or Cisplatin 75mg/m^2^+ Gemcitabine1250mg/m^2^ Q3W)
IMpower131 (NCT02367794) (13)	2020	Phase III,Open Label	IV	343/340	64.0/64.9	557/126	Atezolizumab1200mg Q3W+Chemotherapy (Nab-Paclitaxel 100mg/m^2^+ Carboplatin AUC 6 Q3W)	Chemotherapy (Nab-Paclitaxel 100mg/m^2^ + Carboplatin AUC 6 Q3W)
OAK (NCT02008227) (14)	2017	Phase III,Open Label	IIIB, IV, recurrent	613/612	62.7/62.9	758/467	Atezolizumab 1200 mg Q3W	Chemotherapy (Docetaxel 75mg/m^2^ Q3W)
POPLAR (NCT01903993) (15)	2016	Phase II,Open Label	IIIB, IV, recurrent	144/143	61.5/61.8	169/118	Atezolizumab 1200 mg Q3W	Chemotherapy (Docetaxel 75mg/m^2^ Q3W)
EMPOWER- Lung 1 (NCT03088540) (16)	2021	Phase III,Open Label	IIIB, IV,	356/354	63/64	606/104	Cemiplimab 350mg Q3W	Chemotherapy (Carboplatin AUC 5 or Cisplatin 75mg/m^2^+Gemcitabine1250mg/m^2^ Q3W)
EMPOWER- Lung 3 (NCT03409614) (17)	2022	Phase III	IIIB, IV,	312/154	63/63	391/75	Cemiplimab 350mg Q3W + Chemotherapy(Paclitaxel 200 mg/m^2^+Carboplatin AUC 5 or Cisplatin 75 mg/m^2^)	Chemotherapy(Paclitaxel 200 mg/m^2^+Carboplatin AUC 5 or Cisplatin 75 mg/m^2^)
CameL-sq (NCT03668496) (18)	2021	Phase III	IV	193/196	64/62	359/30	Camrelizumab 200mg Q3W + Chemotherapy (Carboplatin AUC 5 + Paclitaxel 175mg/m^2^ Q3W)	Chemotherapy (Carboplatin AUC 5+Paclitaxel 175mg/m2 Q3W)
POSEIDON (NCT03164616) (19)	2022	Phase III,Open Label	IV	338/337	64.5/64	501/174	Durvalumab1500mg+Chemotherapy(Carboplatin AUC 5 or Cisplatin75mg/m^2^+Gemcitabine1000or1250mg/m^2^ Q3W)	Chemotherapy (Carboplatin AUC 5 or Cisplatin 75mg/m^2^+Gemcitabine1000or1250mg/m^2^ Q3W)
				338/337	63/64	517/158	Durvalumab1500mg+Tremelimumab75mg+Chemotherapy (Carboplatin AUC 5 or Cisplatin 75mg/m^2^+Gemcitabine1000or1250mg/m^2^ Q3W)	
Study 104 (NCT01285609) (20)	2020	Phase III	IV	388/361	64/64	635/114	Ipilimumab 10 mg/kg Q3W +Chemotherapy (Paclitaxel175mg/m²+ Carboplatin AUC6 Q3W)	Placebo+Chemotherapy (Paclitaxel175 mg/m²+ Carboplatin AUC6 Q3W)
CheckMate 227 (NCT02477826) (21)	2019	Phase III,Open Label	IV	583/583	64/64	778/388	Nivolumab 3mg/kg Q2W+ Ipilimumab 1mg /kg Q6W	Chemotherapy(Carboplatin or Cisplatin+Gemcitabine Q3W.)
CheckMate 9LA (NCT03215706) (22)	2021	Phase III	IV	361/358	65/65	504/215	Nivolumab 360mg Q3W + Ipilimumab 1mg/kg Q6W+ Chemotherapy(Paclitaxel200 mg/m²+Carboplatin AUC6 Q3W)	Chemotherapy (Paclitaxel200 mg/m²+ Carboplatin AUC6 Q3W)
CheckMate 017 (NCT01642004) (23)	2017	Phase III, Open Label	IIIB, IV, recurrent	135/137	62/64	208/64	Nivolumab 3 mg/kg Q2W	Chemotherapy (Docetaxel 75mg/m² Q3W )
CheckMate 026 (NCT02041533) (24)	2017	Phase III, Open Label	IV, recurrent	211/212	63/65	332/209	Nivolumab 3 mg/kg Q2W	Chemotherapy(Carboplatin AUC 5 or Cisplatin 75mg/m^2^+ Gemcitabine1000or1250mg/m^2^ Q3W. Paclitaxel 200mg/m^2^+ Carboplatin AUC 6 Q3W)
CheckMate 078 (NCT02613507) (25)	2019	Phase III, Open Label	IIIB, IV	338/166	60/60	397/107	Nivolumab 3 mg/kg Q2W	Chemotherapy (Docetaxel 75mg/m^2^ Q3W)
KEYNOTE-407 (NCT02775435) (26)	2018	Phase III	IV	278/281	65/65	455/104	Pembrolizumab 200mg Q3W +Chemotherapy(Paclitaxel 200mg/m^2^ or nab-paclitaxel 100mg/m^2^ +Carboplatin AUC 6 Q3W)	Placebo+Chemotherapy (Paclitaxel 200mg/m^2^ or nab-paclitaxel 100mg/m^2^ +Carboplatin AUC 6 Q3W)
KEYNOTE-407 China extension studies (NCT03875092) (27)	2021	Phase III	IV	65/60	61.6/61.5	119/6	Pembrolizumab 200mg Q3W +Chemotherapy(Paclitaxel 200mg/m^2^ or nab-paclitaxel 100mg/m^2^ + Carboplatin AUC 6) Q3W)	Placebo+Chemotherapy (Paclitaxel 200mg/m^2^ + Carboplatin AUC 6 Q3W)
KEYNOTE-024 (NCT02142738) (28)	2022	Phase III, Open Label	IV	154/151	64.5/66	187/118	Pembrolizumab 200mg Q3W	Chemotherapy(Paclitaxel 200mg/m2+Carboplatin AUC 6 Q3W.Carboplatin AUC 5 or Cisplatin75mg/m^2^+Gemcitabine 1250mg/m^2^ Q3W.)
KEYNOTE-010 (NCT01905657) (29)	2016	Phase II/III	IIIB, IV, recurrent	344/343	63/62	421/266	Pembrolizumab 2mg/kg Q3W	Chemotherapy (Docetaxel 75mg/m² Q3W )
AK105-302 (NCT03866993) (30)	2024	Phase III	IV	175/175	60.9/61.9	324/26	Penpulimab200mg Q3W +Chemotherapy(Paclitaxel 175mg/m^2^ + Carboplatin AUC 5 Q3W)	Chemotherapy (Paclitaxel 175mg/m^2^ +Carboplatin AUC 5 Q3W)
ORIENT-12 (NCT03629925) (31)	2023	Phase III	IIIB, IV	179/178	64/62	327/30	Sintilimab 200mg Q3W +Chemotherapy (Carboplatin AUC 5 or Cisplatin 75mg/m^2^+ Gemcitabine1250mg/m^2^ Q3W.)	Chemotherapy (Carboplatin AUC 5 or Cisplatin 75mg/m^2^+Gemcitabine 1250mg/m^2^ Q3W.)
ORIENT-3 (NCT03150875) (32)	2023	Phase III, Open Label	IIIB, IV	145/135	61/60	258/22	Sintilimab 200mg Q3W	Chemotherapy (Docetaxel 75mg/m² Q3W )
GEMSTONE- 302 (NCT03789604) (33)	2022	Phase III	IV	320/159	62/64	383/96	Sugemalimab1200mg Q3W + Chemotherapy(Paclitaxel 175mg/m^2^+ Carboplatin AUC 5 Q3W)	Chemotherapy (Paclitaxel 175mg/m^2^+ Carboplatin AUC 5 Q3W)
ASTRUM-004 (NCT04033354) (34)	2023	Phase III	IIIB, IV	358/179	63/63	488/49	Serplulimab 4.5 mg/kg Q3W + Chemotherapy(Carboplatin+Nab-Paclitaxel 100 mg/m^2^ AUC 5 Q3W)	Chemotherapy (Carboplatin+Nab-Paclitaxel100 mg/m^2^ AUC 5 Q3W)
RATIONALE 307 (NCT03594747) (35)	2021	Phase III, Open Label	IIIB, IV	120/121	60/62	218/23	Tislelizumab 200mg Q3W+ Chemotherapy (Paclitaxel 175mg/m2+ Carboplatin AUC 5 Q3W. )	Chemotherapy (Paclitaxel 175mg/m^2^+ Carboplatin AUC 5 Q3W)
RATIONALE 303 (NCT03358875) (36)	2022	Phase III, Open Label	IIIB, IV	535/270	61/61	622/183	Tislelizumab 200mg Q3W	Chemotherapy (Docetaxel 75mg/m^2^)

**Figure 2:**
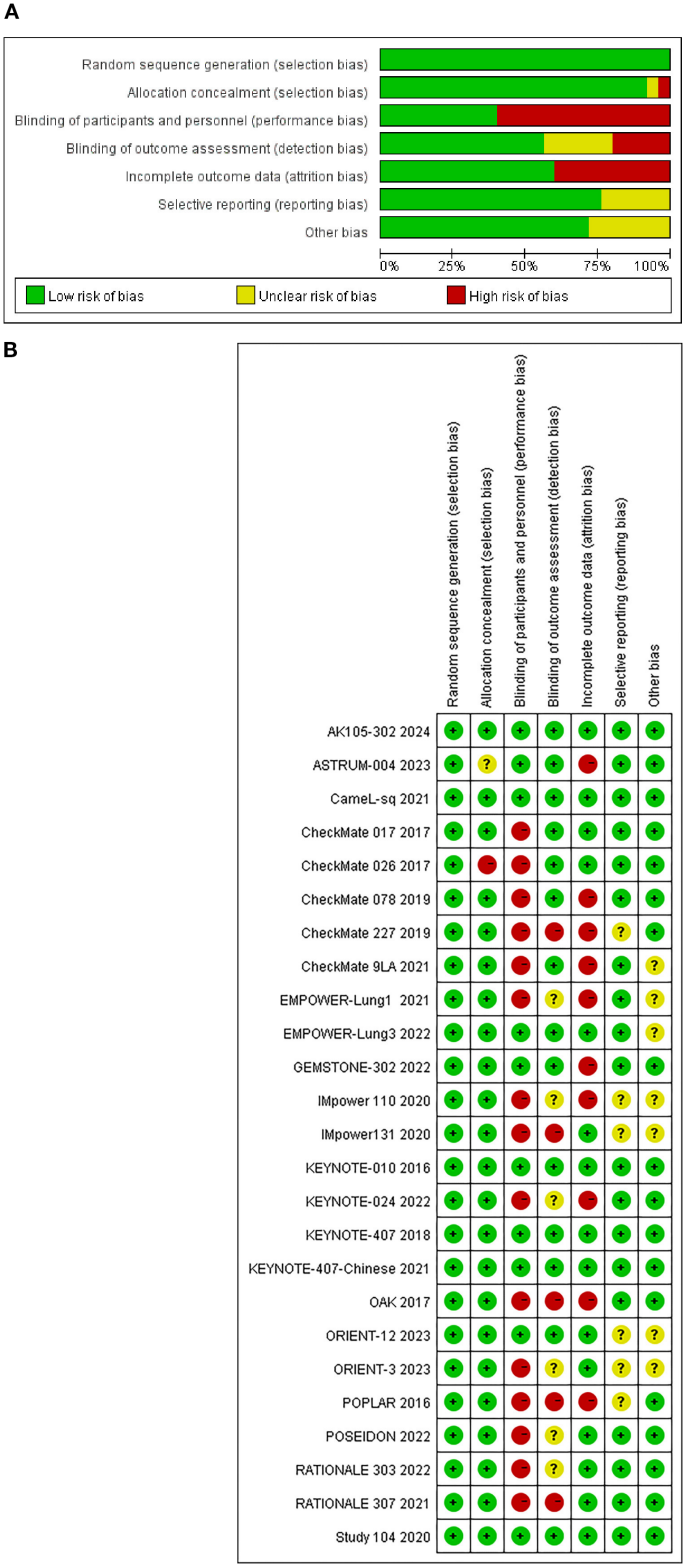
**(A)** Risk of bias graph: review authors’ judgements about each risk of bias item presented as percentages across all included studies. **(B)** Risk of bias summary: review authors’ judgements about each risk of bias item for each included study.

**Figure 2** summarizes the bias risk assessment results, showing that most studies exhibited risks primarily in participant/personnel blinding, outcome assessor blinding, and incomplete outcome data.

The results of the CINeMA assessment are summarized in **Supplementary Table S2**. Of all 190 paired-treatment comparisons, the majority (n = 131, 68.9%) had low confidence and 59 comparisons (31.1%) had very low confidence. No comparisons received moderate or high confidence.

**Figure 3:**
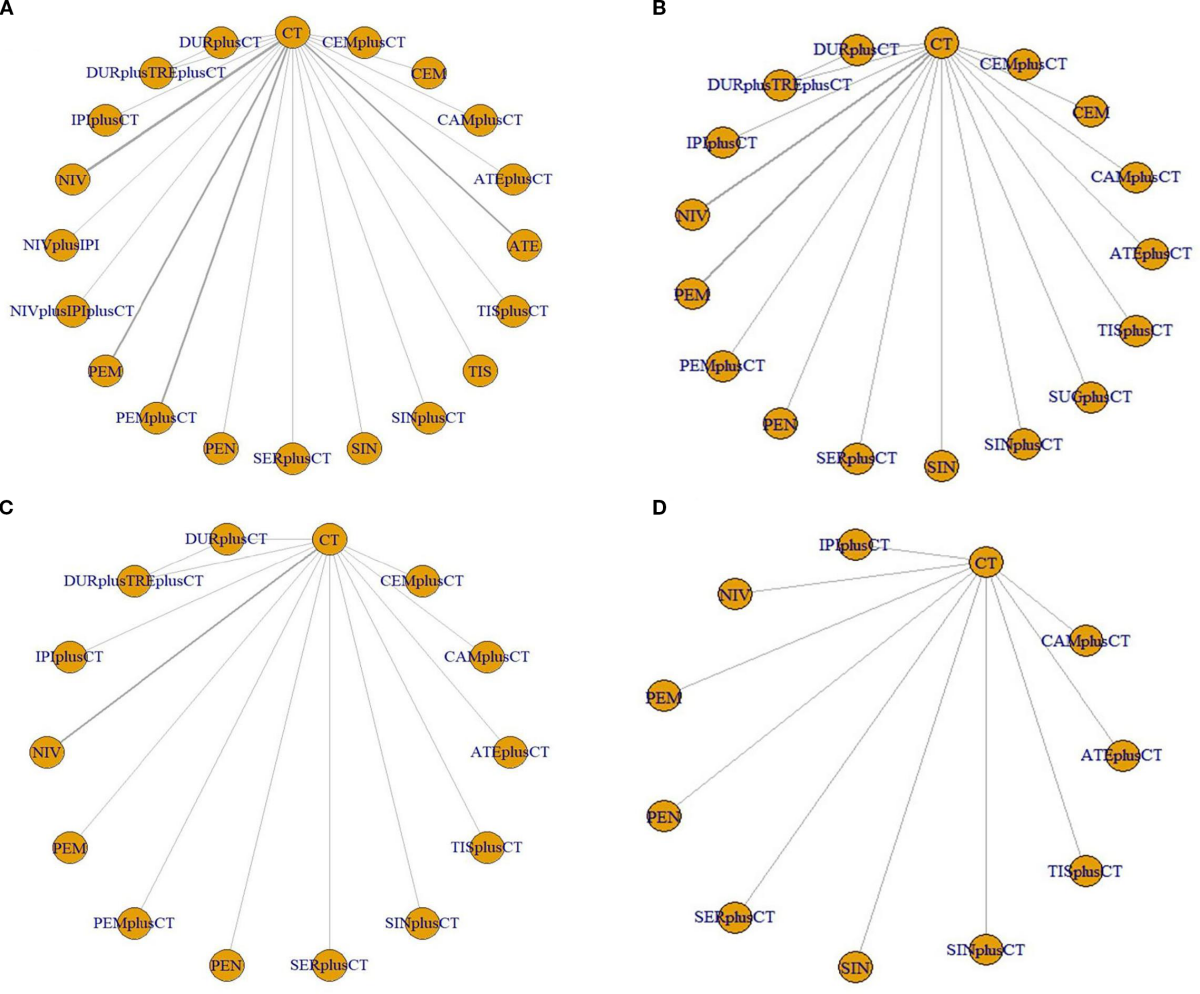
Network evidence plots. **(A)** overall survival **(B)** progression-free survival **(C)** objective response rate **(D)** ≥ grade 3 adverse events. ATE, atezolizumab; ATEplusCT, atezolizumab+chemotherapy; CEM, cemiplimab; CEMplusCT, cemiplimab+chemotherapy; CAMplusCT, camrelizumab+chemotherapy; DURplusCT, durvalumab+chemotherapy; DURplusTREplusCT, durvalumab+tremelimumab+chemotherapy; IPI-CT, ipilimumab+chemotherapy; NIV, nivolumab; NIVplusIPI, nivolumab+ipilimumab; NIVplusIPIplusCT, nivolumab+ipilimumab+chemotherapy; PEM, pembrolizumab; PEM-CT, pembrolizumab+chemotherapy; PEN, penpulimab; SER-CT, serplulimab+chemotherapy; SIN, sintilimab; SIN-CT, sintilimab+chemotherapy; SERplusCT, Serplulimab+chemotherapy; TIS, tislelizumab; TIS-CT, tislelizumab+chemotherapy; CT, chemotherapy.

The study included 21 treatment regimens: chemotherapy, atezolizumab, atezolizumab+chemotherapy, cemiplimab, cemiplimab+chemotherapy,camrelizumab+chemotherapy,durvalumab+chemotherapy,durvalumab+tremelimumab+chemotherapy,ipilimumab+chemotherapy,nivolumab+ipilimumab,nivolumab+ipilimumab+chemotherapy,nivolumab,pembrolizumab,pembrolizumab+chemotherapy,penpulimab+chemotherapy, sintilimab, sintilimab+chemotherapy, sugemalimab, serplulimab+chemotherapy, tislelizumab, tislelizumab+chemotherapy. The network plots are illustrated in **Figure 3**.

### 3.2 Results for overall survival

**Figure 4:**
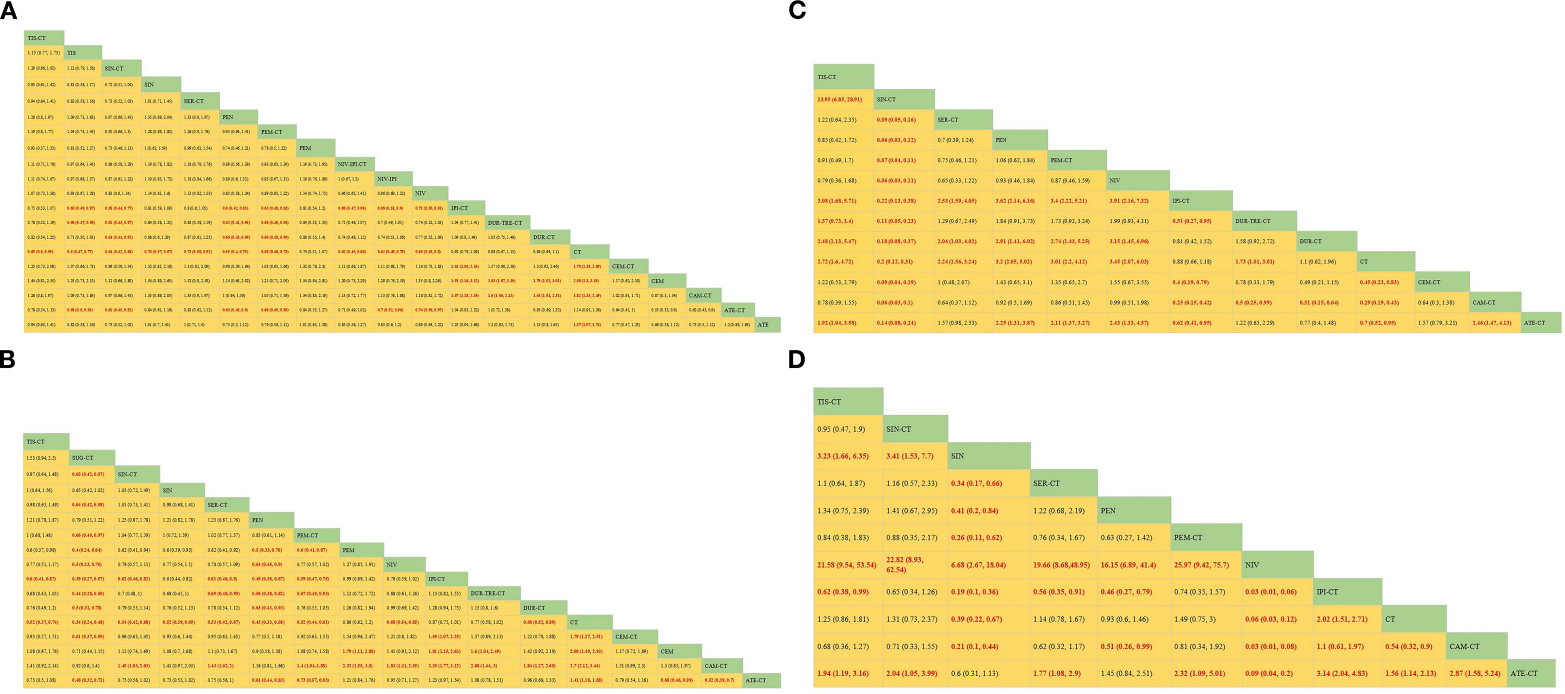
Bayesian network meta-analysis of efficacy and safety metrics in patients with advanced squamous non-small cell lung cancer. **(A)** Hazard ratios and 95% CI for overall survival **(B)** Hazard ratios and 95% CI for progression-free survival, patients with HR< 1.00 had a higher survival rate. **(C)** OR and 95% CI for objective remission rate, OR >1.00 indicates better treatment outcome. **(D)** OR and 95% CI for grade 3 and higher adverse events, OR <1.00 indicates a better safety profile.

**Figure 5:**
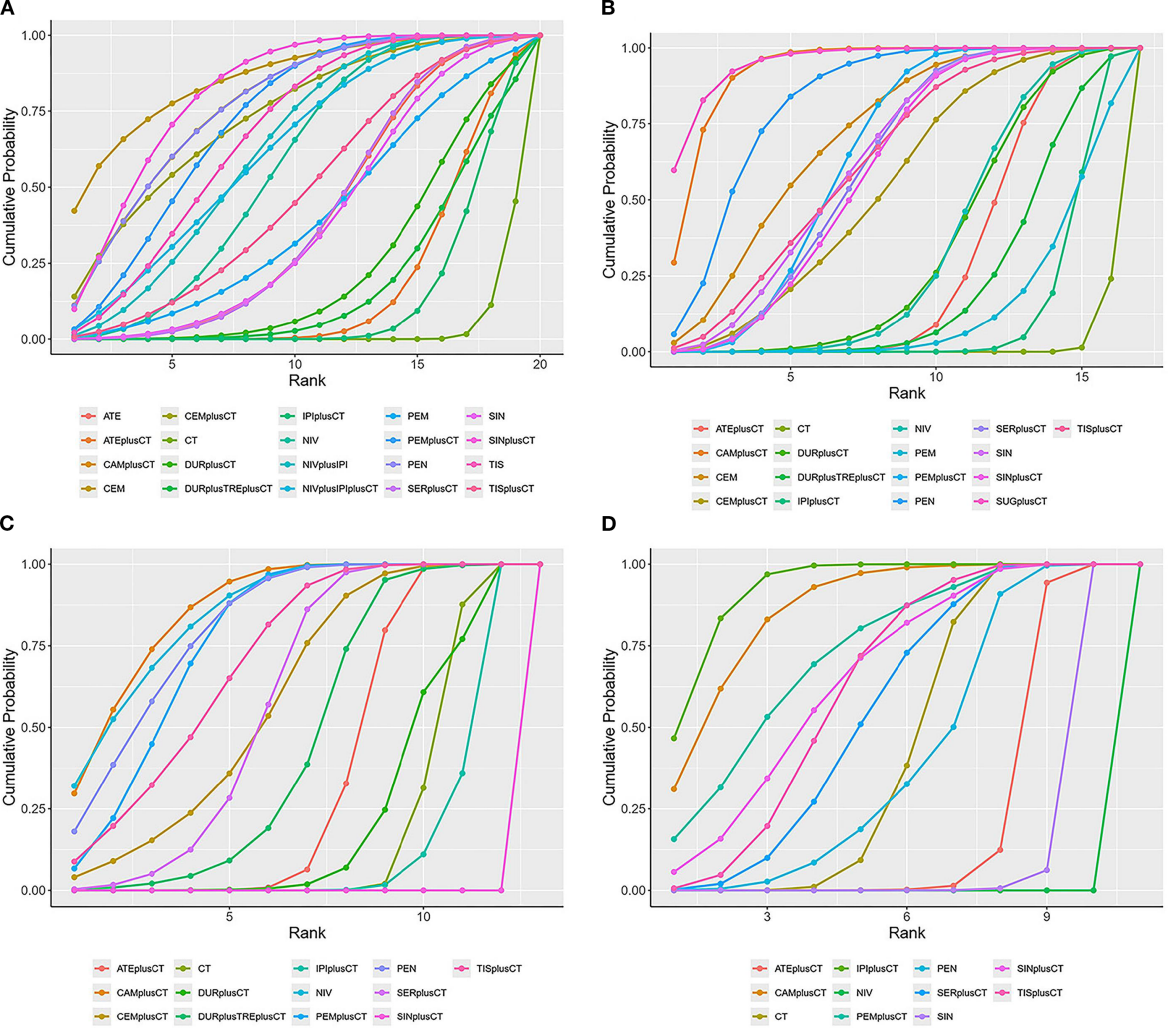
SUCRA cumulative probability ranking plots **(A)** overall survival **(B)** progression-free survival **(C)** objective response rate **(D)** ≥grade 3 adverse events.

Regarding OS (**Figure 4A**), Compared with chemotherapy, except for ipilimumab+chemo [HR = 0.92, 95% CI: (0.59-1.40)], atezolizumab +chemo [HR = 0.88, 95% CI: (0.56-1. 40)], and durvalumab +chemo [HR = 0.84, 95% CI: (0.52-1.40)], durvalumab+tremelimumab+chemo [HR = 0. 88, 95%CI: (0.54-1.40)], which significantly improved OS. Cemiplimab [HR = 0.48, 95% CI: (0.30- 0.77)] had the best OS benefit compared to chemotherapy alone. The SUCRA values of each drug could be analyzed using cumulative probability data according to the Bayesian ordering spectrum (**Figure 5A**). In terms of improving OS in patients with squamous lung cancer, cemiplimab ranked first with a probability of 86.11% of being the drug of choice.

### 3.3 Results for progression free survival

Regarding PFS (**Figure 4B**), immunotherapy significantly increased PFS compared with chemotherapy alone, except for ipilimumab+chemo [HR = 0.87, 95% CI: (0.75-1.00)] and pembrolizumab [HR = 0.86, 95% CI: (0.62-1.20)]. Compared with chemotherapy alone, sugemalimab+chemo [HR = 0.34, 95% CI: (0.24- 0.48)] had the best PFS benefit. According to the Bayesian ranking spectrum, the SUCRA values of each drug could be analyzed using the cumulative probability data (**Figure 5B**), and the probability of sugemalimab+chemo being the preferred drug was ranked first with a probability of 95.45% in improving PFS in patients with squamous lung cancer.

### 3.4 Results for objective response rate

Regarding ORR (**Figure 4C**), immunotherapy significantly improved ORR compared with chemotherapy alone, except for ipilimumab+chemo [OR = 0.88, 95% CI: (0.66-1.20)] and sintilimab+chemo [OR = 0.20, 95% CI: (0.12-0.31)]. Camrelizumab+chemo [OR = 3.50, 95% CI: (2.3- 5.3)] had the best ORR benefit compared with chemotherapy alone. Based on the Bayesian ranking spectrum, the SUCRA values of each drug could be analyzed using cumulative probability data (**Figure 5C**), and the probability of camrelizumab plus chemotherapy being the preferred drug in improving ORR in patients with squamous lung cancer was ranked first with a probability of 86.57%.

### 3.5 Comparisons of safety and toxicity

Comparing safety by analyzing the incidence of grade ≥3 AEs (**Figure 4D**), most immunotherapies had higher rates of adverse events than chemotherapy alone, except for atezolizumab+chemo [OR = 0.64, 95% CI: (0.47-0.88)], nivolumab [OR = 0.06, 95% CI:(0.03-0.12)], and sintilimab [OR = 0.39, 95% CI:(0.22-0.67)], most immunotherapies had a higher rate of grade ≥3 AEs than chemotherapy alone. Based on the Bayesian ranking spectrum, using cumulative probability data the SUCRA values for each drug could be analyzed (**Figure 5D**), and ipilimumab+chemo was most likely (92.66%) to be the most toxic treatment for patients. Adverse reactions greater than or equal to grade 3 frequently reported in immunotherapy combinations included neutropenia, anemia, white blood cell count decreased, platelet count decreased, diarrhea, pneumonia, fatigue. decreased appetite, rash, nausea, asthenia, vomiting, increased ALT, increased AST (**Supplementary Table S2**).

### 3.6 Subgroup analysis

#### 3.6.1 Subgroup analysis of PD-L1 expression levels

We categorized OS and PFS by PD-L1 levels, dividing patients into four groups: <1%, ≥1%, 1%-49%, and ≥50%. Optimal immunotherapy regimens varied for each of the four sub-populations (**Supplementary Figure S1**).

**Figure 6:**
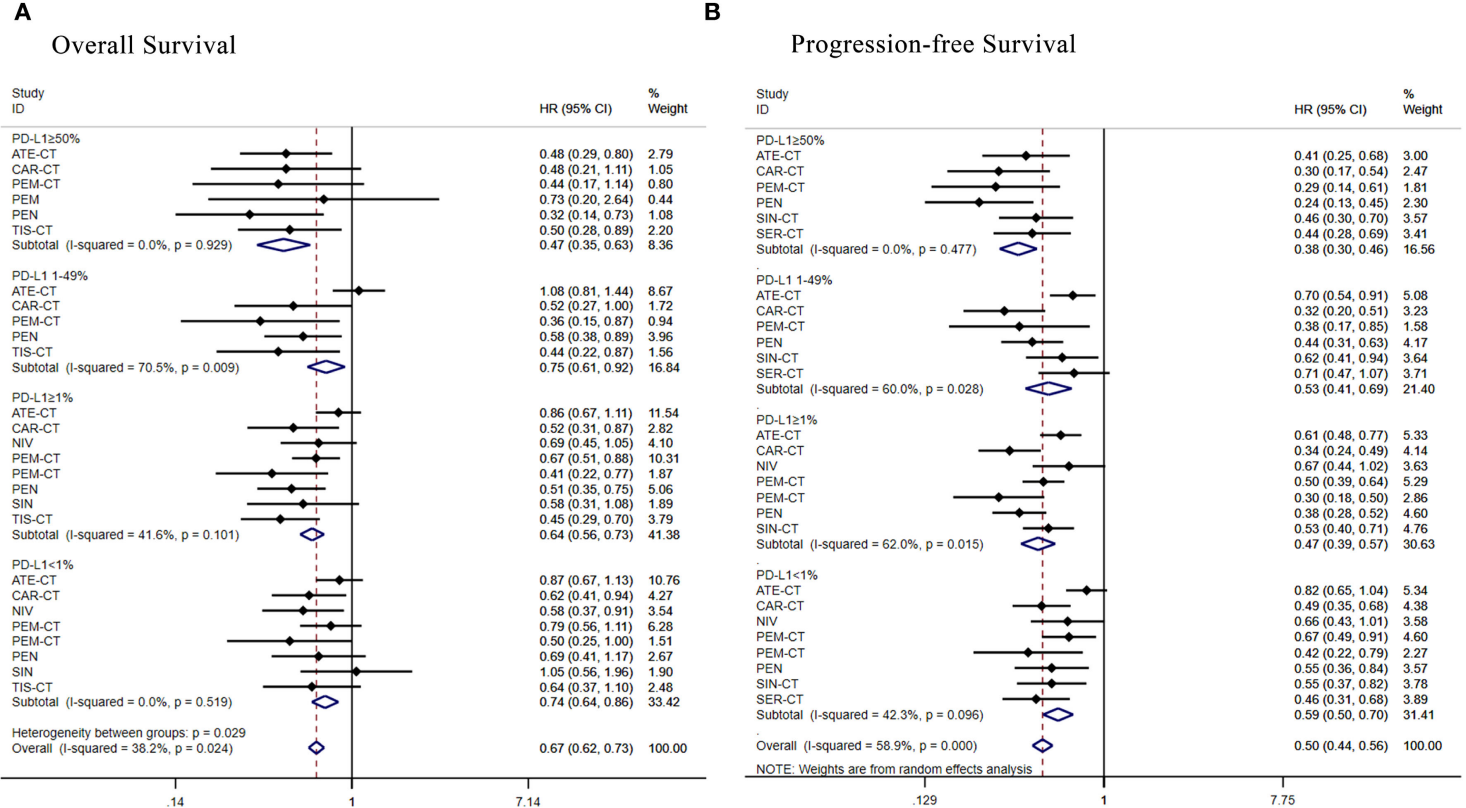
Forest plot of a subgroup analysis of patients with advanced squamous NSCLC according to PD-L1 expression. **(A)** Overall survival **(B)** Progression-free survival.

Eight therapeutic regimens underwent subgroup analysis in PD-L1≥50% patients. Regarding OS (**Figure 6A**), atezolizumab+chemo [HR = 0.48, 95% CI: (0.29-0.80)], camrelizumab+chemo [HR = 0.48, 95% CI:(0. 21-1.11)], pembrolizumab+chemo[HR = 0.44, 95% CI: (0.17-1.14)], pembrolizumab [HR = 0.73, 95% CI: (0.20-2.64)], penpulimab [HR = 0.32, 95% CI: 0.14-0.73)] and tislelizumab+chemo [HR = 0.47, 95% CI: (0.35-0. 63)] were superior to chemotherapy alone. As for PFS (**Figure 6B**), atezolizumab+chemo[HR = 0.41,95%CI:(0.25-0.68)],camrelizumab+chemo[HR 0.30, 95% CI:(0.17-0.54)], pembrolizumab+chemo [HR = 0.29, 95% CI:(0.14-0.61)],penpulimab[HR = 0.24,95%CI:(0.13-0.45)],sintilimab+chemo[HR = 0.46, 95%CI:(0.30-0.70)] and serplulimab+chemo [HR = 0.44, 95% CI: (0.28-0.69)] were superior to chemotherapy alone.

Seven regimens were assessed in the PD-L1 1%-49% subgroup. Regarding OS (**Figure 6A**), camrelizumab+chemo [HR = 0.52, 95% CI: (0.27-1.00)], pembrolizumab+chemo [HR = 0.36, 95% CI: (0.15-0.87)], penpulimab [HR = 0.58, 95% CI: (0.38-0.89)] and tislelizumab+chemo [HR = 0.44, 95% CI: (0.22-0.87)] were superior to chemotherapy alone, while atezolizumab+chemo [HR = 1.08, 95% CI: (0.81- 1.44)] failed to benefit compared to chemotherapy alone. As for PFS (**Figure 6B**), atezolizumab+chemo[HR = 0.70,95%CI:(0.54-0.91)],camrelizumab+chemo[HR = 0.32,95%CI:(0.20-0.51)],pembrolizumab+chemo [HR = 0.38, 95% CI: (0.17-0.85)], penpulimab[HR = 0.44,95%CI:(0.31-0.63)], sintilimab+chemo[HR = 0.62, 95% CI: (0.41-0.9)] and serplulimab+chemo [HR = 0.71, 95% CI: (0.47-1.07)]were more favorable than chemotherapy.

Subgroup analysis of PD-L1≥1% patients evaluated eight regimens. Regarding OS (**Figure 6A**), camrelizumab+chemo [HR = 0.52, 95% CI: (0.31-0.87)],nivolumab[HR = 0.69,95%CI:(0.45-1.05)],pembrolizumab+chemo [HR = 0.67, 95% CI: (0.51-0.88)], pembrolizumab+chemo [HR = 0.41, 95% CI:(0.22-0.77)], penpulimab [HR = 0.51, 95% CI: (0.22-0.77)], pembrolizumab+chemo [HR = 0.41, 95% CI: (0.22-0.77)], penpulimab [HR = 0.51, 95% CI: (0.35-0.75)], sintilimab [HR = 0.58, 95% CI: (0. 31-1.08)], tislelizumab+chemo [HR = 0.45, 95% CI: (0.29-0.70)] were superior to chemotherapy alone, while atezolizumab+chemo [HR = 0. 86, 95% CI: (0.67-1.11)] failed to benefit. As for PFS (**Figure 6B**), atezolizumab+chemo[HR = 0.61,95%CI:(0.48-0.77)],camrelizumab+chemo [HR = 0.34, 95% CI: (0.24-0.49)], nivolumab [HR = 0.67, 95% CI: 0.44-1.02)], pembrolizumab+chemo [HR = 0.50, 95% CI: (0.39-0.64)], pembrolizumab+chemo [HR = 0.30, 95% CI: (0.18-0.50)], penpulimab [HR = 0.38, 95% CI: (0.28-0.52)], sintilimab+chemo [HR = 0.53, 95% CI: (0.40-0.71)] were superior to chemotherapy alone over standard chemotherapy.

Subgroup analysis of PD-L1<1% patients evaluated eight regimens. Regarding OS (**Figure 6A**), camrelizumab+chemo [HR = 0.62, 95% CI: 0.41-0.94)],nivolumab[HR = 0.58,95%CI:(0.37-0.91)],pembrolizumab+ chemo [HR = 0.79, 95% CI: (0.56-1.11)], pembrolizumab+chemo [HR = 0.50, 95% CI: (0. 25-1. 00)], penpulimab [HR = 0.69, 95% CI: (0. 41-1. 17)] and tislelizumab+chemo [HR = 0.64, 95% CI: (0. 37-1.10)] were superior to chemotherapy alone, while atezolizumab+chemo [HR = 0.87, 95% CI: (0.67-1.13)], and sintilimab+chemo [HR = 1.05, 95% CI: (0.56-1.96)] failed to benefit. As for PFS (**Figure 6B**), camrelizumab+chemo [HR = 0. 49, 95% CI: (0. 35-0. 68)], nivolumab [HR = 0. 66, 95% CI: (0. 43-1. 01)], and pembrolizumab+chemo [HR = 0. 67, 95% CI: (0. 49-0. 91)], pembrolizumab +chemo [HR = 0.42, 95% CI: (0.22-0.79)],penpulimab[HR = 0.55,95%CI:(0.36-0.84)],sintilimab+chemo [HR = 0.55, 95% CI: (0.37-0.82)] were superior to chemotherapy alone over standard chemotherapy, while atezolizumab+chemo [HR = 0.82, 95% CI: (0.65-1.04)] failed to benefit.

#### 3.6.2 Subgroup analysis of therapeutic lines

We grouped OS and PFS according to the number of treatment lines and categorized patients into first- and second-line therapy. Optimal immunotherapy regimens vary between first- and second-line treatments (**Supplementary Figure S2**).

**Figure 7:**
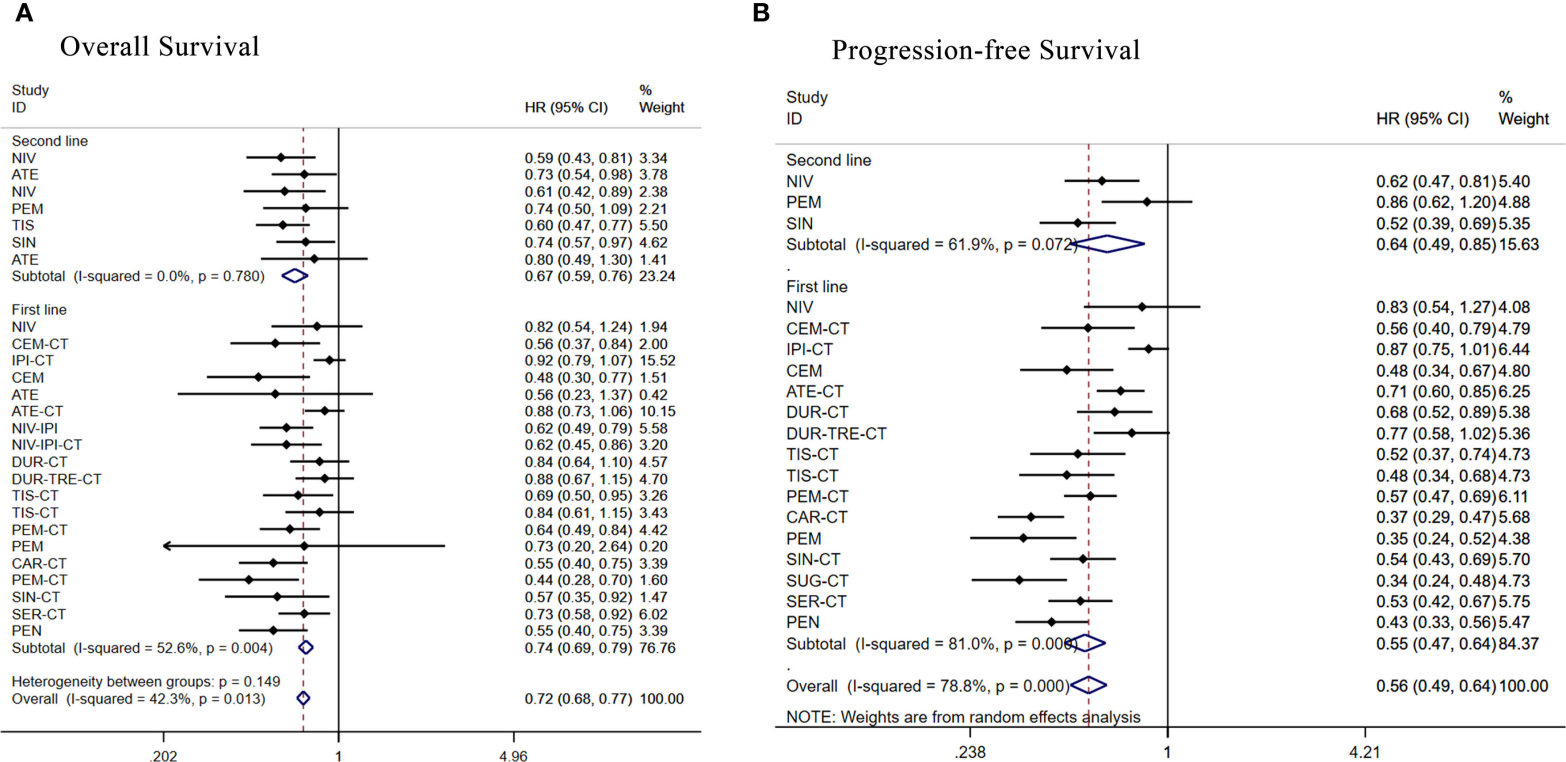
Forest plot of subgroup analysis of patients with advanced squamous NSCLC according to treatment line **(A)** overall survival **(B)** progression-free survival.

There were 19 RCTs as first-line treatment. Regarding OS (**Figure 7A**), apart from ipilimumab+chemo [HR = 0.92, 95% CI: (0.79-1.07)], atezolizumab+chemo[HR = 0.88,95%CI:(0.73-1.06)],durvalumab+tremelimumab+chemo [HR = 0.88, 95% CI: (0.68-1.16)], durvalumab+chemo [HR = 0.84, 95% CI: (0.64-1.10)], tislelizumab+chemo [HR = 0.84, 95% CI: (0.61-1.14)], and nivolumab[HR = 0.82, 95% CI: (0.54-1.24)], all other treatment regimens demonstrated significant OS benefits. Regarding PFS (**Figure 7B**), all treatment regimens except ipilimumab+chemo [HR = 0.87, 95% CI: (0.75-1.01)] and nivolumab [HR = 0.83, 95% CI: (0.54-1.26)]showed better PFS benefit.

Seven RCTs were included in second-line treatment. Regarding OS (**Figure 7A**), the remaining treatment regimens had better OS benefit except for atezolizumab [HR = 0.80, 95% CI: (0.49-1.30)]. Regarding PFS (**Figure 7B**), the remaining treatment regimens had better PFS benefit except for pembrolizumab [HR = 0.86, 95% CI: (0.62-1.20)].

#### 3.6.3 Subgroup analysis of race

We grouped OS and PFS according to ethnicity, categorizing patients into Asian and non-Asian. The optimal immunotherapy regimen varied by ethnicity (**Supplementary Figure S3**).

**Figure 8:**
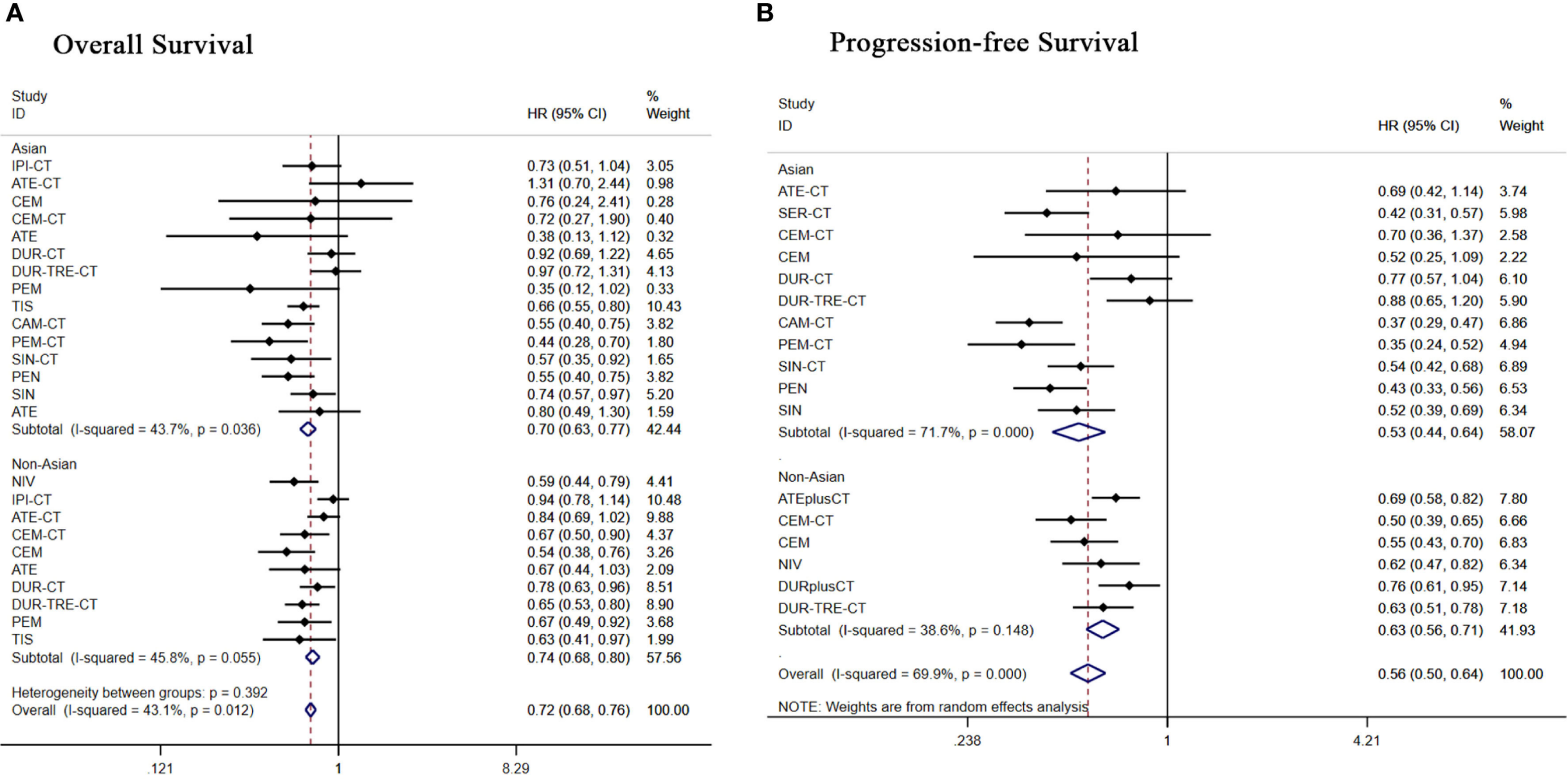
Forest plot of subgroup analysis of patients with advanced squamous NSCLC according to race **(A)** overall survival **(B)** progression-free survival.

Among Asian patients, 15 treatment regimens were subgrouped and analyzed. Regarding OS (**Figure 8A**), apart from durvalumab+tremelimumab+chemo [HR = 0.97, 95% CI: (0.72-1.31)], durvalumab+chemo [HR = 0.92, 95% CI: (0.69-1.22)], all other treatment regimens demonstrated significant OS benefits. Regarding PFS (**Figure 8B**), all treatment regimens except durvalumab+tremelimumab+chemo [HR = 0.88, 95% CI: (0.65-1.20)]showed better PFS benefit.

Subgroup analysis of 10 treatment regimens in non-Asian patients. Regarding OS (**Figure 8A**), apart from ipilimumab+chemo[HR = 0.94, 95% CI: (0.78-1.14)], atezolizumab+chemo[HR = 0.84,95%CI:(0.69-1.02)], all other treatment regimens demonstrated significant OS benefits. Regarding PFS (**Figure 8B**), durvalumab+chemo [HR = 0.76, 95% CI: (0.61-0.95)], all other treatment regimens showed significant PFS benefit.

#### 3.6.4 Subgroup analysis of smoking history

We grouped patients for OS and PFS based on their smoking history and categorized patients as former/current smokers and never smokers. The optimal immunotherapy regimen varied among patients with different smoking histories (**Supplementary Figure S4**).

**Figure 9:**
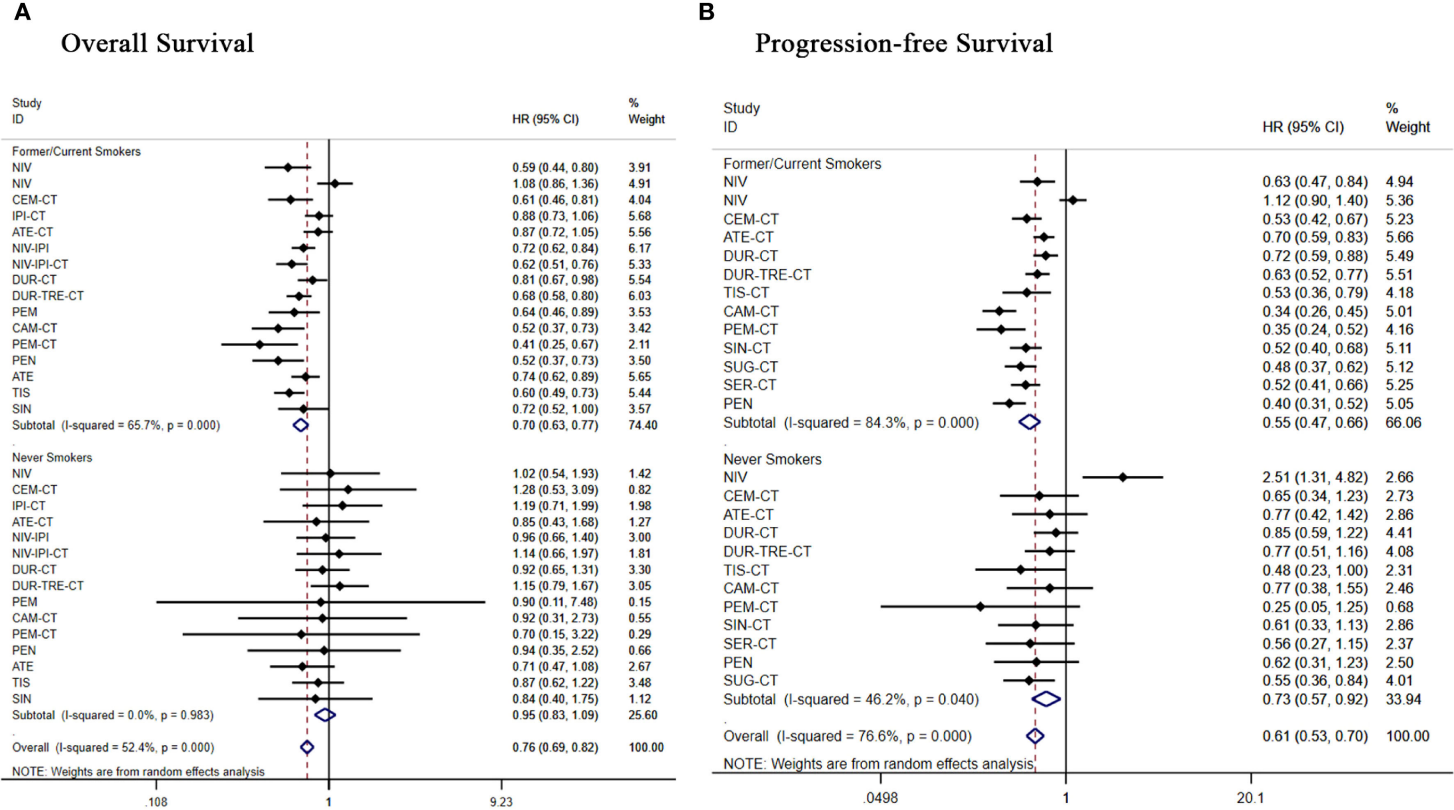
Forest plot of a subgroup analysis of patients with advanced squamous NSCLC according to smoking history **(A)** overall survival **(B)** progression-free survival.

Subgroup analysis of 19 treatment regimens in former or current smokers. Regarding OS (**Figure 9A**), apart from NIV [HR = 1.08, 95% CI: (0.86-1.36)], ipilimumab+chemo[HR = 0.88, 95% CI: (0.73-1.06)], atilizumab+chemo[HR = 0.87, 95% CI: (0.72-1.05)] and durvalumab+chemo [HR = 0.81, 95% CI: (0.67-0.98)], all other treatment regimens showed significant OS benefit. Regarding PFS (**Figure 9B**), all treatment regimens except nivolumab [(HR = 1.12, 95% CI: 0.90-1.40)] showed significant PFS benefit.

Subgroup analysis of 17 treatment regimens for never smokers. In terms of OS (**Figure 9A**), only pembrolizumab+chemo [(HR = 0.70, 95% CI: 0.15-3.22)] and atezolizumab [(HR = 0.71, 95% CI: 0.47-1.08)] demonstrated an overall survival benefit in never smokers, all other regimens did not show significant improvement in OS. In terms of PFS (**Figure 9B**), all regimens showed significant improvement in PFS except nivolumab [(HR = 2.51, 95% CI:1.31-4.82)], atezolizumab+chemo [(HR = 0.77, 95% CI: 0.42-1.42)], durvalumab+chemo [(HR = 0.85, 95% CI: 0.59-1.22)], durvalumab+tremelimumab+chemo [(HR = 0.77, 95% CI: 0.51-1.16)], camrelizumab+chemo[(HR = 0.77, 95% CI:0.38-1.55)], which showed significant improvement in PFS.

### 3.7 Convergence, inconsistency, and publication bias assessment

Our computational results stemmed from a model set-up with 4 Markov chains, a 10-step size and 20,000 annealing iterations, then 50,000 sampling cycles. Convergence was evaluated via historical data, ensuring stable and reproducible MCMC inferences. **Supplementary Figures S3**, **S4** display the results. We also assessed study heterogeneity using the Q-test and I^2^ statistic, which showed low heterogeneity of included studies (**Supplementary Figure S5**). Funnel plot analysis showed that there was no significant publication bias for OS, whereas there was a potential publication bias for PFS (**Supplementary Figure S6**). Our assessment using the Egger test showed (**Supplementary Figure S7**) that there was no statistically significant publication bias for either endpoint (P > 0.05).

## 4 Discussion

Targeting immune checkpoints has redefined NSCLC management over the past ten years, establishing immunotherapy as a cornerstone treatment. Nivolumab and pembrolizumab obtained regulatory authorization for second-line clinical application in 2015, supported by phase III trial evidence confirming survival advantages over standard chemotherapy regimens. In 2016, the PD-L1 inhibitor atezolizumab gained approval for this NSCLC indication (37). The success of ICIs in second-line therapy has rapidly spurred research into first-line treatment. In KEYNOTE-024, pembrolizumab significantly outperformed chemotherapy in both PFS and OS outcomes (28). In 2016, pembrolizumab became the first ICI approved for the treatment of NSCLC patients with a PD-L1 TPS of ≥ 50% (37). Following this, regulatory approvals expanded to include nivolumab, atezolizumab, and other PD-1/PD-L1 inhibitors for first-line use. Currently, ongoing phase III trials are exploring novel ICI combinations, including dual checkpoint blockade and the combination of immunotherapy with targeted agents (38). This rapid clinical translation reflects the great potential and remaining challenges of immunotherapy in NSCLC, especially in terms of optimal patient selection and overcoming resistance mechanisms.

A recent meta-analysis by Li and Sorin et al. (39, 40) comprehensively demonstrated the superiority of neoadjuvant chemotherapy combined with immunotherapy over chemotherapy alone in terms of multiple prognostic indicators in a broad population of NSCLC patients. This study systematically compared various immunotherapy regimens in first- and second-line treatments for advanced lung squamous cell carcinoma patients, including immunotherapy monotherapy, immunotherapy combined with chemotherapy, and dual immunotherapy strategies. Directly addressing the clinical decision-making needs for patients with advanced, unresectable lung squamous cell carcinoma, this study fills the evidence gap in comparing immunotherapy strategies for this specific population. Additionally, we included more recent clinical trial studies to provide a more comprehensive ranking of treatment options and comparisons of their advantages and disadvantages. This study precisely focuses on the treatment population of squamous non-small cell lung cancer, conducting in-depth and systematic subgroup analyses, including different population characteristics, smoking history, and PD-L1 expression levels. It not only reveals the efficacy differences of immunotherapy across different subgroups but also provides evidence for personalized treatment. By employing Bayesian network meta-analysis to overcome the limitations of traditional direct comparisons, we comprehensively quantified the relative efficacy rankings of various immunotherapy combination regimens, offering a more comprehensive assessment. Our study provides the inaugural assessment of therapeutic regimens based on immunotherapy for driver-negative advanced squamous NSCLC, using network meta-analysis to evaluate both effectiveness and toxicity profiles. We found that immune-related therapy was able to provide significant benefit to the total population of advanced wild-type squamous lung cancer relative to chemotherapy alone. Of these, Cemiplimab was superior to Chemo and any other incorporated therapeutic agent in terms of OS benefit. The NCCN guidelines designate cemiplimab as the preferred initial therapy for individuals diagnosed with squamous NSCLC exhibiting PD-L1 expression levels of 50% or higher (41). In terms of PFS, sugilizumab had superior PFS benefit than chemotherapy and any other incorporated therapeutic agents. Sugemalimab (CS1001) acts as a fully human, full-length immunoglobulin G4 (IgG4) monoclonal antibody targeting PD-L1 with the S228P hinge-stabilizing mutation. Preclinical studies demonstrate sugemalimab’s preserved Fcγ receptor I (FcγRI) binding capacity drives PD-L1+ tumor cell phagocytosis via macrophage engagement, while potentially augmenting tumor antigen cross-presentation (42, 43). In the GEMSTONE-302 study, particularly in the squamous subgroup, sugemalimab demonstrated a better PFS benefit (33). Among the treatments, camrelizumab plus chemotherapy provided the optimal ORR advantage. Camrelizumab (SHR-1210), a PD-1-targeting humanized IgG4 monoclonal antibody, demonstrates clinically significant antitumor activity and favorable safety profiles across multiple malignancies, particularly in lung cancer (44–48).

We noted that ipilimumab plus chemotherapy did not lead to better efficacy, which may be due to insufficient activation of effector T cells, and although ipilimumab plus chemotherapy promotes the activation of early T cells in the lymphoid region, this mechanism does not seem to be sufficient to generate a strong antitumor response in squamous NSCLC (49). However, concomitant use of ipilimumab and nivolumab improved clinical efficacy thanks to complementary mechanisms in dual immune checkpoint blockade. This dual-targeted strategy may enhance tumor cell eradication through complementary mechanisms: ipilimumab promotes T-cell activation and proliferation, while nivolumab supports existing T-cell recognition and targeting of tumor cells. Crucially, ipilimumab-primed lymphocytes acquire memory phenotypes, enabling sustained tumor surveillance (50). Dual-immunization regimens are more effective than monoimmunotherapy through dual-targeted inhibition of both the immune activation and immune effect phases. Based on this mechanistic advantage, the FDA approved the nivolumab-ipilimumab-chemotherapy triple combination regimen for advanced NSCLC in 2020 based on critical clinical evidence (21).

In advanced SqNSCLC cases where PD-L1 is ≥50%, penpulimab provided significant survival benefit, with OS and PFS superior to conventional chemotherapy regimens. Meanwhile, patients with low PD-L1-negative or PD-L1-negative had little clinical benefit from atezolizumab + chemotherapy, but patients with high PD-L1 had a better survival benefit. Patients with PD-L1 1-49% demonstrated optimal OS with pembrolizumab+chemo and superior PFS with camrelizumab+chemo; those with PD-L1 ≥1% achieved optimal OS and PFS using either tislelizumab or camrelizumab+chemo; while for PD-L1-negative tumors (<1%), nivolumab+chemo and serplulimab+chemo provided the best OS and PFS outcomes.

High PD-L1 expression should theoretically enhance the effect of PD-1/PD-L1 blockade. However, in patients with PD-L1≥50%, we observed that pembrolizumab monotherapy failed to demonstrate a significant OS benefit, while penpulimab monotherapy yielded the most favorable OS outcomes. Additionally, pembrolizumab+chemo exhibited superior OS benefit compared to pembrolizumab monotherapy. This suggests a limitation of PD-L1 as a single biomarker in guiding immunotherapy selection for advanced squamous lung cancer. The significant differences in clinical efficacy of different ICIs highlight the importance of the drug’s own properties (e.g., antibody structure, target affinity, etc.). Pembrolizumab is a humanized IgG4 monoclonal antibody that specifically targets PD-1, exerting its antitumor effect by competitively inhibiting PD-L1 binding through high-affinity interactions between its CDR regions and PD-1’s CC’FG β-sheet and BC/C’D/FG loop structures (51). Penpulimab is a novel humanized anti-PD-1 antibody and is the only IgG1 subtype anti-PD-1 monoclonal antibody with a modified fragment crystallizable segment. Penpulimab has a more stable structure, lower antigen-binding dissociation rate, and higher receptor occupancy rate than other anti-PD-1 drugs that might have the potential to improve efficacy and safety (52).

In addition to PD-L1 expression, metrics such as TMB, mismatch repair defects, microsatellite instability, tumor-infiltrating lymphocytes, and intestinal microbiota have demonstrated unique value in the prediction of efficacy and prognostic assessment of immunotherapy. It is necessary to establish a comprehensive evaluation system that can integrate multidimensional biomarkers, and provide a more accurate basis for individualized treatment decisions by comprehensively analyzing the tumor characteristics and immune status of patients, so as to ultimately maximize the clinical benefits for patients.

We analyzed subgroups of patients by ethnicity. A subgroup analysis of Asian patients showed that several immunotherapy regimens including tislelizumab, pembrolizumab+chemotherapy, camrelizumab+ chemotherapy, sintilimab, pembrolizumab and Serplulimab+ chemotherapy showed potential survival benefit in Asian patients compared to chemotherapy alone. Asian patients had the best OS and PFS with pembrolizumab or pembrolizumab + chemotherapy. Significantly improved survival was observed in non-Asians receiving nivolumab, cemiplimab, durvalumab+tremelimumab, or durvalumab+chemotherapy compared to chemotherapy. Non-Asian patients achieved the best OS and PFS with cemiplimab. Subgroup analyses by ethnicity revealed important insights into the efficacy of immunotherapy for advanced squamous NSCLC, highlighting the potential benefits of certain regimens, and pointing to the need for further research to optimize treatment strategies based on patient ethnicity.

Subgroup analysis of immunotherapy outcomes in patients with advanced squamous NSCLC stratified by smoking history. The analysis showed that among former/current smokers, all immunologic regimens except nivolumab demonstrated significant survival benefit compared to chemotherapy. Among them, pembrolizumab+chemotherapy and Camrelizumab + chemotherapy had the best OS and PFS. Among never smokers, most immunization regimens did not significantly improve OS and PFS in nonsmokers; nivolumab even significantly increased the risk of progression.

This may be due to the fact that carcinogens in tobacco smoke significantly increase somatic mutational load in lung cancer. Studies have shown that NSCLC in smokers typically has a high tumor mutation burden (TMB), and that carcinogens in tobacco consistently induce DNA damage, leading to mutation accumulation. Tumors of smokers with high TMB produce more *de novo* antigens, which are recognized by T cells and thus enhance the immune system’s response to the tumor. In contrast, lung cancers in nonsmokers typically have a low TMB and are often accompanied by mutations in driver genes such as EGFR resulting in limited efficacy of PD-1 inhibitors (53).

Immunotherapy serves as a critical component of second-line therapy for patients whose disease progresses following initial chemotherapy. In second-line regimens where all therapies were administered as monotherapy, we observed PD-1 inhibitors demonstrated superior efficacy to PD-L1 inhibitors. This different effect may stem from the unique mechanism of PD-1 inhibitors, which block receptor signaling simultaneously by preventing engagement with PD-L1 and PD-L2 ligands. In contrast, PD-L1 inhibitors only block the PD-L1/PD-1 interaction while preserving the PD-L2-mediated immunosuppressive signaling through PD-1 receptors, potentially allowing residual immune escape mechanisms (54). PD-1 inhibitors may activate T cell function more broadly, whereas PD-L1 inhibitors are more dependent on tumor cell or stromal cell PD-L1 (55).

Treatment-related grade≥3 adverse events occurred more frequently with immunotherapy versus chemotherapy alone. Furthermore, our findings suggest that monotherapy immunotherapy is less likely to induce high-grade adverse events and demonstrates a safer profile compared to chemoimmunotherapy combinations. Among immunotherapies, the Ipilimumab plus chemotherapy regimen exhibited the highest toxicity burden. The irAEs may be linked to ICIs altering systemic immune homeostasis. Mechanistically, the CTLA-4 pathway exerts suppressive effects during the priming phase of T-cell responses by modulating T-cell activation in lymph nodes and altering regulatory T-cell (Treg) function. Consequently, CTLA-4 inhibitors are associated with broader-spectrum irAEs characterized by higher incidence rates, lower specificity, and more severe toxicity compared to other ICIs (56).

Common Grade ≥3 related toxicities in immunotherapy are primarily hematologic toxicity, gastrointestinal toxicity, fatigue, decreased appetite, pneumonitis, dermatotoxicity, and hepatic function abnormalities. When diarrhea-immunization-related adverse events of grade 3 or higher occur, ICIs therapy should be suspended, intravenous methylprednisolone should be used, and the addition of Infliximab should be considered along with continued hormone application. Suspend ICIs therapy with concomitant topical administration of a potent steroid such as Prednisolone when an adverse event rash of grade 3 or greater occurs. Permanently discontinue ICIs therapy when a serious immune-related adverse event pneumonitis occurs involving all lobes of the lungs or >50% of the lung parenchyma, with personal self-care limited and requiring oxygenation. If infection has not been completely ruled out, empiric anti-infective therapy is required. After 48 hours of intravenous Methylprednisolone hormone therapy, if clinical symptoms improve, continue therapy until symptoms improve to ≤ G1. If no significant improvement is seen, consider receiving infliximab IV (57–60).The management of irAEs should be carried out throughout the treatment process, including prevention, examination, assessment, treatment and monitoring. Before the start of treatment, baseline examinations, such as laboratory indicators, imaging and organ function assessment, should be completed to provide a control basis for the subsequent judgment of symptoms. If new onset or worsening of symptoms occurs during treatment, physical examination, laboratory and imaging evaluations, combined with the patient’s underlying disease and baseline data, should be used to differentiate irAEs from disease progression or other episodic events. irAEs can occur at any stage of immunotherapy, and the timing of their onset correlates with the organ involved. Regular post-treatment monitoring of symptoms and index changes is required, as well as prevention of opportunistic infections, calcium supplementation, and gastric management during hormone use. For restarting immunotherapy after remission of irAEs, the severity of the last event, the patient’s general condition, and the alternative treatment regimen should be taken into consideration. After restarting immunotherapy, close monitoring is needed, especially focusing on the functional status of previously involved organs, to ensure the safety of the treatment (61).

This study has limitations: first, some of the trials were limited to specific populations and ethnic groups, such as only Chinese patients or people with high PD-L1 expression, which may lead to population heterogeneity. Second, most randomized controlled trials included squamous NSCLC as an outcome of subgroup analyses, and some studies did not report safety outcomes for this subgroup separately, resulting in a lack of comprehensive data for network meta-analyses of grade ≥3 adverse events and distinct toxicity profiles. Future prospective phase III clinical trials are needed for advanced SqNSCLC to directly compare different immune checkpoint inhibitor regimens. Third, and most fundamentally, the comparative conclusions regarding efficacy and safety are based on a sparsely connected network. This sparsity manifests as a lack of direct head-to-head comparisons between many related interventions, leading to most inferences being highly reliant on indirect comparisons. While these indirect comparisons are valuable, they are uncertain compared to the direct evidence from randomized trials, and the sparsity of the evidence network thus limits its clinical applicability. This highlights the need for more clinical trials in advanced SqNSCLC in the future, especially head-to-head studies that directly compare different immune checkpoint inhibitor regimens and combination strategies, thus providing stronger comparative evidence for optimal regimen selection. In addition, the synergistic advancement of a standardized adverse events reporting system based on histological subtype stratification and cross-ethnic cohort studies will provide a key support for the construction of a precise clinical decision support system for these patients.

## 5 Conclusion

Immune-related therapy can provide significant benefit relative to chemotherapy alone in advanced SqNSCLC, both first- and second-line. PD-L1 expression level affects immunotherapy outcomes: for PD-L1 ≥50%, penpulimab had favorable OS and PFS outcomes; for PD-L1 1-49%, pembrolizumab + chemo was superior for OS benefit and camrelizumab + chemo was superior for PFS benefit; and for patients with PD-L1 ≥1%, the tislelizumab + chemo had positive efficacy in OS and camrelizumab + chemo had positive efficacy in PFS; and for patients with PD-L1 <1%, both nivolumab and serplulimab + chemo provided significant survival benefit. For Asian patients, those with pembrolizumab or pembrolizumab + chemotherapy had excellent OS and PFS benefit. For non-Asian patients had a favorable benefit with cemiplimab. In former/current smokers, pembrolizumab+chemotherapy and camrelizumab+chemotherapy had significant OS and PFS benefit, but in never smokers, most immunotherapies did not significantly improve OS and PFS. Compared to existing guidelines (e.g., NCCN), this study adds treatment options. We included strong evidence for multiple novel and effective regimens, including penpulimab, camrelizumab, tislelizumab, serplulimab, sintilimab, and sugemalimab, all of which have demonstrated superior clinical benefit. At the same time this work provides refined, evidence-based optimized regimens for challenging subgroups, such as proven effective regimens (nivolumab, serplulimab + chemo) for the historically difficult-to-treat PD-L1<1% population where guideline choices are most limited. It is important to emphasize that clinical decisions should be made in the context of individual patient characteristics, including comorbidities, drug accessibility, and tolerability. We systematically evaluated survival outcomes and toxicity profiles of first- and second-line immune checkpoint inhibitor monotherapy and combination therapy in SqNSCLC, stratified by PD-L1 expression level, ethnicity, and smoking history. Evidence-based medical evidence for precision interventions in different populations is provided through the development of biomarker-based therapeutic decision-making models to optimize long-term survival benefit and therapeutic safety management for patients.

## Data Availability

The original contributions presented in the study are included in the article/**Supplementary Material**. Further inquiries can be directed to the corresponding author.
