# Supplementary material for: Efficacy and safety of immune checkpoint inhibitors for advanced squamous non-small cell lung cancer: a systematic review and network meta-analysis
Source: Front Immunol. 2025 Sep 16;16:1635757. doi: 10.3389/fimmu.2025.1635757 (PMC12479475; doi:10.3389/fimmu.2025.1635757)
Supplement: Supplementary file 1 [file Table1.docx]

Supplementary Table 1. Search Strategies

(((((((((((((((((((((((((((((((((((("Carcinoma, non-Small Cell"[Mesh]) ) OR (Carcinomas, Small Cell[Title/Abstract])) OR (Small Cell Carcinomas[Title/Abstract])) OR (Carcinoma, Oat Cell[Title/Abstract])) OR (Carcinomas, Oat Cell[Title/Abstract])) OR (Oat Cell Carcinomas[Title/Abstract])) OR (Oat Cell Carcinoma[Title/Abstract])) OR (Small Cell Carcinoma[Title/Abstract])) AND (chemotherapy[Title/Abstract])) OR (pemetrexed[Title/Abstract])) OR (Gemcitabine[Title/Abstract])) OR (Docetaxel[Title/Abstract])) OR (Cisplatin[Title/Abstract])) OR (carboplatin[Title/Abstract])) OR (platinum[Title/Abstract])) OR (Paclitaxel[Title/Abstract])) OR (Paclitaxel-albumin[Title/Abstract])) OR (nab-paclitaxel[Title/Abstract])) OR (Abraxane[Title/Abstract])) OR (Gemcitabine[Title/Abstract])) OR ((((((((((("Nivolumab"[Mesh]) OR (MDX-1106[Title/Abstract])) OR (MDX1106[Title/Abstract])) OR (MDX 1106[Title/Abstract])) OR (Opdivo[Title/Abstract])) OR (BMS-936558[Title/Abstract])) OR (BMS936558[Title/Abstract])) OR (BMS 936558[Title/Abstract])) OR (ONO-4538[Title/Abstract])) OR (ONO4538[Title/Abstract])) OR (ONO 4538[Title/Abstract]))) OR ((((("pembrolizumab" [Supplementary Concept]) OR (MK-3475[Title/Abstract])) OR (Keytruda[Title/Abstract])) OR (lambrolizumab[Title/Abstract])) OR (SCH-900475[Title/Abstract]))) OR (((((((("atezolizumab" [Supplementary Concept]) OR (immunoglobulin G1, anti-(human CD antigen CD274) (human monoclonal MDPL3280a heavy chain), disulfide with human monoclonal MDPL3280a kappa-chain, dimer[Title/Abstract])) ) OR (MPDL3280A[Title/Abstract])) OR (MPDL-3280A[Title/Abstract])) OR (Tecentriq[Title/Abstract])) OR (RG7446[Title/Abstract])) OR (RG-7446[Title/Abstract]))) OR (((((((((("Ipilimumab"[Mesh]) OR (Anti-CTLA-4 MAb Ipilimumab[Title/Abstract])) OR (Anti CTLA 4 MAb Ipilimumab[Title/Abstract])) OR (Ipilimumab, Anti-CTLA-4 MAb[Title/Abstract])) OR (MDX 010[Title/Abstract])) OR (MDX-010[Title/Abstract])) OR (MDX010[Title/Abstract])) OR (MDX-CTLA-4[Title/Abstract])) OR (MDX CTLA 4[Title/Abstract])) OR (Yervoy[Title/Abstract]))) OR (("cemiplimab" [Supplementary Concept]) OR (REGN2810[Title/Abstract]))) OR ((((((((("tremelimumab" [Supplementary Concept]) OR (CP-675,206[Title/Abstract])) OR (CP 675206[Title/Abstract])) OR (CP-675206[Title/Abstract])) OR (CP675206[Title/Abstract])) OR (CP 675[Title/Abstract])) OR (CP-675[Title/Abstract])) OR (CP675 cpd[Title/Abstract])) OR (Ticilimumab[Title/Abstract]))) OR (((("durvalumab" [Supplementary Concept]) OR (MEDI4736[Title/Abstract])) OR (MEDI-4736[Title/Abstract])) OR (Imfinzi[Title/Abstract]))) OR (("tislelizumab" [Supplementary Concept]) OR (BGB-A317[Title/Abstract]))) OR (((("camrelizumab" [Supplementary Concept]) OR (SHR-1210[Title/Abstract])) OR (SHR 1210[Title/Abstract])) OR (Carrelizumab[Title/Abstract]))) OR (((("sintilimab" [Supplementary Concept]) OR (IBI 308[Title/Abstract])) OR (IBI308[Title/Abstract])) OR (IBI-308[Title/Abstract]))) OR ((((("necitumumab" [Supplementary Concept]) OR (Portrazza[Title/Abstract])) OR (IMC-11F8[Title/Abstract])) OR (IMC-11F8 monoclonal antibody[Title/Abstract])) OR (11F8[Title/Abstract]))) OR (Sugemalimab[Title/Abstract])) OR (Penpulimab[Title/Abstract])) OR ((((((((((((((("Immunotherapy"[Mesh]) OR (Immune checkpoint[Title/Abstract])) OR (immunotherapy[Title/Abstract])) OR (Programmed cell death 1 ligand 1[Title/Abstract])) OR (Programmed cell death 1[Title/Abstract])) OR (Programmed cell death 1 Receptor[Title/Abstract])) OR (Programmed cell death 1 ligand 1 protein[Title/Abstract])) OR (antiPDL1[Title/Abstract])) OR (antiPD1[Title/Abstract])) OR (PD-1[Title/Abstract])) OR (PD 1[Title/Abstract])) OR (PD1[Title/Abstract])) OR (PD-L1[Title/Abstract])) OR (PD L1[Title/Abstract])) OR (PDL1[Title/Abstract]))) ) AND (randomized clinical trials[Title/Abstract])

Supplementary Table 2. Summary table for credibility assessment using CINeMA.

| **Comparison** | **Within-study bias** | **Reporting bias** | **Indirectness** | **Imprecision** | **Heterogeneity** | **Incoherence** | **Confidence rating** |
| --- | --- | --- | --- | --- | --- | --- | --- |
| ATEvsATE-CT |  |  |  |  |  |  | Very low |
| ATEvsCAM-CT |  |  |  |  |  |  | Low |
| ATEvsCEM |  |  |  |  |  |  | Very low |
| ATEvsCEM-CT |  |  |  |  |  |  | Very low |
| ATEvsDUR-CT |  |  |  |  |  |  | Low |
| ATEvsDUR-TRE-CT |  |  |  |  |  |  | Low |
| ATEvsIPI-CT |  |  |  |  |  |  | Low |
| ATEvsNIV |  |  |  |  |  |  | Low |
| ATEvsNIV-IPI |  |  |  |  |  |  | Very low |
| ATEvsNIV-IPI-CT |  |  |  |  |  |  | Very low |
| ATEvsPEM |  |  |  |  |  |  | Low |
| ATEvsPEM-CT |  |  |  |  |  |  | Low |
| ATEvsPEN |  |  |  |  |  |  | Low |
| ATEvsSER-CT |  |  |  |  |  |  | Very low |
| ATEvsSIN |  |  |  |  |  |  | Very low |
| ATEvsSIN-CT |  |  |  |  |  |  | Very low |
| ATEvsSUG-CT |  |  |  |  |  |  | Very low |
| ATEvsTIS |  |  |  |  |  |  | Very low |
| ATEvsTIS-CT |  |  |  |  |  |  | Very low |
| ATE-CTvsCAM-CT |  |  |  |  |  |  | Low |
| ATE-CTvsCEM |  |  |  |  |  |  | Very low |
| ATE-CTvsCEM-CT |  |  |  |  |  |  | Very low |
| ATE-CTvsDUR-CT |  |  |  |  |  |  | Low |
| ATE-CTvsDUR-TRE-CT |  |  |  |  |  |  | Low |
| ATE-CTvsIPI-CT |  |  |  |  |  |  | Low |
| ATE-CTvsNIV |  |  |  |  |  |  | Low |
| ATE-CTvsNIV-IPI |  |  |  |  |  |  | Very low |
| ATE-CTvsNIV-IPI-CT |  |  |  |  |  |  | Very low |
| ATE-CTvsPEM |  |  |  |  |  |  | Low |
| ATE-CTvsPEM-CT |  |  |  |  |  |  | Low |
| ATE-CTvsPEN |  |  |  |  |  |  | Low |
| ATE-CTvsSER-CT |  |  |  |  |  |  | Very low |
| ATE-CTvsSIN |  |  |  |  |  |  | Very low |
| ATE-CTvsSIN-CT |  |  |  |  |  |  | Very low |
| ATE-CTvsSUG-CT |  |  |  |  |  |  | Very low |
| ATE-CTvsTIS |  |  |  |  |  |  | Very low |
| ATE-CTvsTIS-CT |  |  |  |  |  |  | Very low |
| CAM-CTvsCEM |  |  |  |  |  |  | Low |
| CAM-CTvsCEM-CT |  |  |  |  |  |  | Low |
| CAM-CTvsDUR-CT |  |  |  |  |  |  | Low |
| CAM-CTvsDUR-TRE-CT |  |  |  |  |  |  | Low |
| CAM-CTvsIPI-CT |  |  |  |  |  |  | Low |
| CAM-CTvsNIV |  |  |  |  |  |  | Low |
| CAM-CTvsNIV-IPI |  |  |  |  |  |  | Low |
| CAM-CTvsNIV-IPI-CT |  |  |  |  |  |  | Low |
| CAM-CTvsPEM |  |  |  |  |  |  | Low |
| CAM-CTvsPEM-CT |  |  |  |  |  |  | Low |
| CAM-CTvsPEN |  |  |  |  |  |  | Low |
| CAM-CTvsSER-CT |  |  |  |  |  |  | Low |
| CAM-CTvsSIN |  |  |  |  |  |  | Low |
| CAM-CTvsSIN-CT |  |  |  |  |  |  | Low |
| CAM-CTvsSUG-CT |  |  |  |  |  |  | Low |
| CAM-CTvsTIS |  |  |  |  |  |  | Low |
| CAM-CTvsTIS-CT |  |  |  |  |  |  | Low |
| CEMvsCEM-CT |  |  |  |  |  |  | Low |
| CEMvsDUR-CT |  |  |  |  |  |  | Low |
| CEMvsDUR-TRE-CT |  |  |  |  |  |  | Low |
| CEMvsIPI-CT |  |  |  |  |  |  | Low |
| CEMvsNIV |  |  |  |  |  |  | Low |
| CEMvsNIV-IPI |  |  |  |  |  |  | Very low |
| CEMvsNIV-IPI-CT |  |  |  |  |  |  | Very low |
| CEMvsPEM |  |  |  |  |  |  | Low |
| CEMvsPEM-CT |  |  |  |  |  |  | Low |
| CEMvsPEN |  |  |  |  |  |  | Low |
| CEMvsSER-CT |  |  |  |  |  |  | Very low |
| CEMvsSIN |  |  |  |  |  |  | Very low |
| CEMvsSIN-CT |  |  |  |  |  |  | Low |
| CEMvsSUG-CT |  |  |  |  |  |  | Very low |
| CEMvsTIS |  |  |  |  |  |  | Low |
| CEMvsTIS-CT |  |  |  |  |  |  | Very low |
| CEM-CTvsDUR-CT |  |  |  |  |  |  | Low |
| CEM-CTvsDUR-TRE-CT |  |  |  |  |  |  | Low |
| CEM-CTvsIPI-CT |  |  |  |  |  |  | Low |
| CEM-CTvsNIV |  |  |  |  |  |  | Low |
| CEM-CTvsNIV-IPI |  |  |  |  |  |  | Very low |
| CEM-CTvsNIV-IPI-CT |  |  |  |  |  |  | Very low |
| CEM-CTvsPEM |  |  |  |  |  |  | Low |
| CEM-CTvsPEM-CT |  |  |  |  |  |  | Low |
| CEM-CTvsPEN |  |  |  |  |  |  | Low |
| CEM-CTvsSER-CT |  |  |  |  |  |  | Low |
| CEM-CTvsSIN |  |  |  |  |  |  | Low |
| CEM-CTvsSIN-CT |  |  |  |  |  |  | Low |
| CEM-CTvsSUG-CT |  |  |  |  |  |  | Low |
| CEM-CTvsTIS |  |  |  |  |  |  | Low |
| CEM-CTvsTIS-CT |  |  |  |  |  |  | Low |
| DUR-CTvsIPI-CT |  |  |  |  |  |  | Low |
| DUR-CTvsNIV |  |  |  |  |  |  | Low |
| DUR-CTvsNIV-IPI |  |  |  |  |  |  | Low |
| DUR-CTvsNIV-IPI-CT |  |  |  |  |  |  | Low |
| DUR-CTvsPEM |  |  |  |  |  |  | Low |
| DUR-CTvsPEM-CT |  |  |  |  |  |  | Low |
| DUR-CTvsPEN |  |  |  |  |  |  | Very low |
| DUR-CTvsSER-CT |  |  |  |  |  |  | Low |
| DUR-CTvsSIN |  |  |  |  |  |  | Low |
| DUR-CTvsSIN-CT |  |  |  |  |  |  | Low |
| DUR-CTvsSUG-CT |  |  |  |  |  |  | Low |
| DUR-CTvsTIS |  |  |  |  |  |  | Low |
| DUR-CTvsTIS-CT |  |  |  |  |  |  | Low |
| DUR-TRE-CTvsIPI-CT |  |  |  |  |  |  | Low |
| DUR-TRE-CTvsNIV |  |  |  |  |  |  | Low |
| DUR-TRE-CTvsNIV-IPI |  |  |  |  |  |  | Low |
| DUR-TRE-CTvsNIV-IPI-CT |  |  |  |  |  |  | Low |
| DUR-TRE-CTvsPEM |  |  |  |  |  |  | Low |
| DUR-TRE-CTvsPEM-CT |  |  |  |  |  |  | Low |
| DUR-TRE-CTvsPEN |  |  |  |  |  |  | Low |
| DUR-TRE-CTvsSER-CT |  |  |  |  |  |  | Low |
| DUR-TRE-CTvsSIN |  |  |  |  |  |  | Low |
| DUR-TRE-CTvsSIN-CT |  |  |  |  |  |  | Low |
| DUR-TRE-CTvsSUG-CT |  |  |  |  |  |  | Low |
| DUR-TRE-CTvsTIS |  |  |  |  |  |  | Low |
| DUR-TRE-CTvsTIS-CT |  |  |  |  |  |  | Low |
| IPI-CTvsNIV |  |  |  |  |  |  | Low |
| IPI-CTvsNIV-IPI |  |  |  |  |  |  | Low |
| IPI-CTvsNIV-IPI-CT |  |  |  |  |  |  | Low |
| IPI-CTvsPEM |  |  |  |  |  |  | Low |
| IPI-CTvsPEM-CT |  |  |  |  |  |  | Low |
| IPI-CTvsPEN |  |  |  |  |  |  | Low |
| IPI-CTvsSER-CT |  |  |  |  |  |  | Low |
| IPI-CTvsSIN |  |  |  |  |  |  | Low |
| IPI-CTvsSIN-CT |  |  |  |  |  |  | Low |
| IPI-CTvsSUG-CT |  |  |  |  |  |  | Low |
| IPI-CTvsTIS |  |  |  |  |  |  | Low |
| IPI-CTvsTIS-CT |  |  |  |  |  |  | Low |
| NIVvsNIV-IPI |  |  |  |  |  |  | Low |
| NIVvsNIV-IPI-CT |  |  |  |  |  |  | Low |
| NIVvsPEM |  |  |  |  |  |  | Low |
| NIVvsPEM-CT |  |  |  |  |  |  | Low |
| NIVvsPEN |  |  |  |  |  |  | Low |
| NIVvsSER-CT |  |  |  |  |  |  | Low |
| NIVvsSIN |  |  |  |  |  |  | Low |
| NIVvsSIN-CT |  |  |  |  |  |  | Low |
| NIVvsSUG-CT |  |  |  |  |  |  | Low |
| NIVvsTIS |  |  |  |  |  |  | Low |
| NIVvsTIS-CT |  |  |  |  |  |  | Low |
| NIV-IPIvsNIV-IPI-CT |  |  |  |  |  |  | Very low |
| NIV-IPIvsPEM |  |  |  |  |  |  | Low |
| NIV-IPIvsPEM-CT |  |  |  |  |  |  | Low |
| NIV-IPIvsPEN |  |  |  |  |  |  | Low |
| NIV-IPIvsSER-CT |  |  |  |  |  |  | Very low |
| NIV-IPIvsSIN |  |  |  |  |  |  | Very low |
| NIV-IPIvsSIN-CT |  |  |  |  |  |  | Very low |
| NIV-IPIvsSUG-CT |  |  |  |  |  |  | Very low |
| NIV-IPIvsTIS |  |  |  |  |  |  | Very low |
| NIV-IPIvsTIS-CT |  |  |  |  |  |  | Very low |
| NIV-IPI-CTvsPEM |  |  |  |  |  |  | Low |
| NIV-IPI-CTvsPEM-CT |  |  |  |  |  |  | Low |
| NIV-IPI-CTvsPEN |  |  |  |  |  |  | Low |
| NIV-IPI-CTvsSER-CT |  |  |  |  |  |  | Very low |
| NIV-IPI-CTvsSIN |  |  |  |  |  |  | Very low |
| NIV-IPI-CTvsSIN-CT |  |  |  |  |  |  | Very low |
| NIV-IPI-CTvsSUG-CT |  |  |  |  |  |  | Very low |
| NIV-IPI-CTvsTIS |  |  |  |  |  |  | Very low |
| NIV-IPI-CTvsTIS-CT |  |  |  |  |  |  | Very low |
| PEMvsPEM-CT |  |  |  |  |  |  | Low |
| PEMvsPEN |  |  |  |  |  |  | Low |
| PEMvsSER-CT |  |  |  |  |  |  | Low |
| PEMvsSIN |  |  |  |  |  |  | Low |
| PEMvsSIN-CT |  |  |  |  |  |  | Low |
| PEMvsSUG-CT |  |  |  |  |  |  | Low |
| PEMvsTIS |  |  |  |  |  |  | Low |
| PEMvsTIS-CT |  |  |  |  |  |  | Low |
| PEM-CTvsPEN |  |  |  |  |  |  | Low |
| PEM-CTvsSER-CT |  |  |  |  |  |  | Low |
| PEM-CTvsSIN |  |  |  |  |  |  | Low |
| PEM-CTvsSIN-CT |  |  |  |  |  |  | Low |
| PEM-CTvsSUG-CT |  |  |  |  |  |  | Low |
| PEM-CTvsTIS |  |  |  |  |  |  | Low |
| PEM-CTvsTIS-CT |  |  |  |  |  |  | Low |
| PENvsSER-CT |  |  |  |  |  |  | Low |
| PENvsSIN |  |  |  |  |  |  | Low |
| PENvsSIN-CT |  |  |  |  |  |  | Low |
| PENvsSUG-CT |  |  |  |  |  |  | Low |
| PENvsTIS |  |  |  |  |  |  | Low |
| PENvsTIS-CT |  |  |  |  |  |  | Low |
| SER-CTvsSIN |  |  |  |  |  |  | Low |
| SER-CTvsSIN-CT |  |  |  |  |  |  | Low |
| SER-CTvsSUG-CT |  |  |  |  |  |  | Low |
| SER-CTvsTIS |  |  |  |  |  |  | Low |
| SER-CTvsTIS-CT |  |  |  |  |  |  | Low |
| SINvsSIN-CT |  |  |  |  |  |  | Low |
| SINvsSUG-CT |  |  |  |  |  |  | Low |
| SINvsTIS |  |  |  |  |  |  | Low |
| SINvsTIS-CT |  |  |  |  |  |  | Low |
| SIN-CTvsSUG-CT |  |  |  |  |  |  | Low |
| SIN-CTvsTIS |  |  |  |  |  |  | Low |
| SIN-CTvsTIS-CT |  |  |  |  |  |  | Low |
| SUG-CTvsTIS |  |  |  |  |  |  | Low |
| SUG-CTvsTIS-CT |  |  |  |  |  |  | Low |
| TISvsTIS-CT |  |  |  |  |  |  | Low |

ATE:atezolizumab;CAM:camrelizumab;CEM:cemiplimab;DUR:durvalumab;TRE:tremelimumab;IPI:ipilimumab;NIV:nivolumab;PEM: pembrolizumab;PEN:penpulimab;SER:erplulimab;SIN:sintilimab;SUG:sugemalimab;TIS:tislelizumab;CT:chemotherapy.

Supplementary Table 3. Number of Grade ≥3 Adverse Events with Higher Rates of Each Immunotherapy

|  | NIV | IPI-CT | CAM-CT | SIN-CT | SER-CT | PEN | SIN | ATE-CT | PEM-CT | TIS-CT |
| --- | --- | --- | --- | --- | --- | --- | --- | --- | --- | --- |
| Neutropenia | 0 | 18 | 107 | 87 | 53 | 77 | 0 | 25 | 64 | 62 |
| Anaemia | 0 | 29 | 20 | 60 | 43 | 3 | 1 | 31 | 44 | 9 |
| White blood cell count decreased | 1 | 0 | 58 | 65 | 36 | 35 | 0 | 6 | NR | 27 |
| Platelet count decreased | 0 | 17 | 13 | 81 | 24 | 6 | 0 | 14 | 23 | 5 |
| Diarrhea | 0 | 27 | 4 | NR | 0 | 0 | NR | 6 | 12 | NR |
| Pneumonia | 0 | NR | 7 | 25 | 5 | NR | 1 |  | 9 | NR |
| Fatigue | 1 | 15 | NR | NR | 2 | 2 | 1 | 8 | 13 | NR |
| Decreased appetite | 1 | 17 | 1 | 1 | 1 | NR | NR | 2 | 7 | 1 |
| Rash | 0 | 17 | 1 | 3 | 1 | 1 | 2 | 2 | 2 | 4 |
| Nausea | 0 | 16 | 0 | 2 | 2 | 1 | 0 | 3 | 4 | 0 |
| Asthenia | 0 | 7 | 1 | 3 | 3 | NR | NR | 10 | 6 | 0 |
| Vomiting | 0 | 9 | 1 | 4 | 3 | 0 | NR | 1 | 1 | 1 |
| Increased ALT | 0 | 6 | 3 | 1 | 4 | NR | 4 | NR | NR | 2 |
| Increased AST | 0 | 5 | 1 | 1 | 2 | 4 | 2 | NR | NR | 0 |

ATE-CT:atezolizumab+chemotherapy;CAM-CT:camrelizumab+chemotherapy;IPI-CT:ipilimumab+chemotherapy;NIV:nivolumab;PEM-CT:pembrolizumab+chemotherapy;PEN:penpulimab;SER-CT:serplulimab+chemotherapy;SIN:sintilimab;SIN-CT:sintilimab+chemotherapy;TIS-CT:tislelizumab+chemotherapy;NR:no report;ALT:alanine aminotransferase;AST:aspartate aminotransferase.


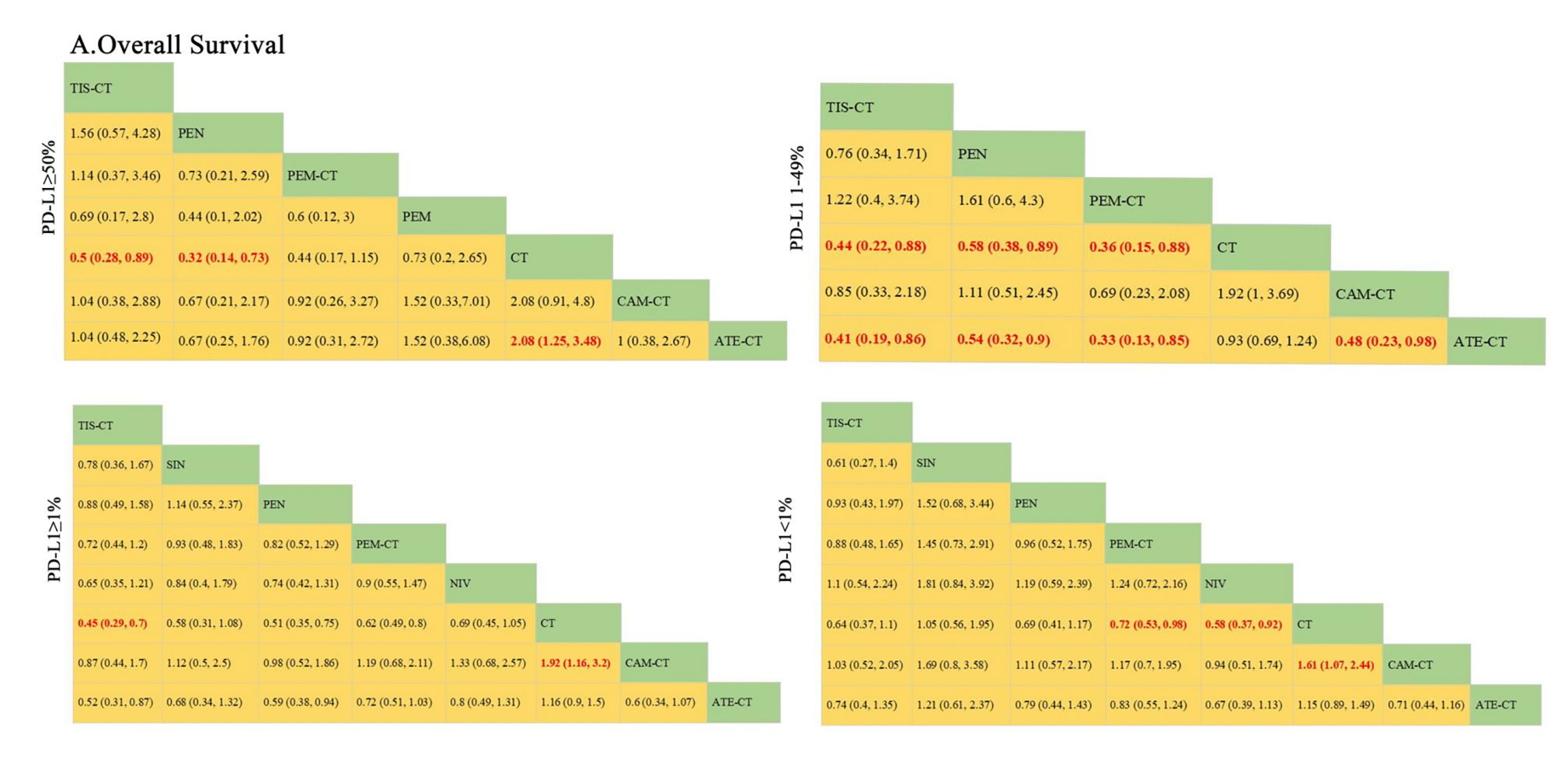


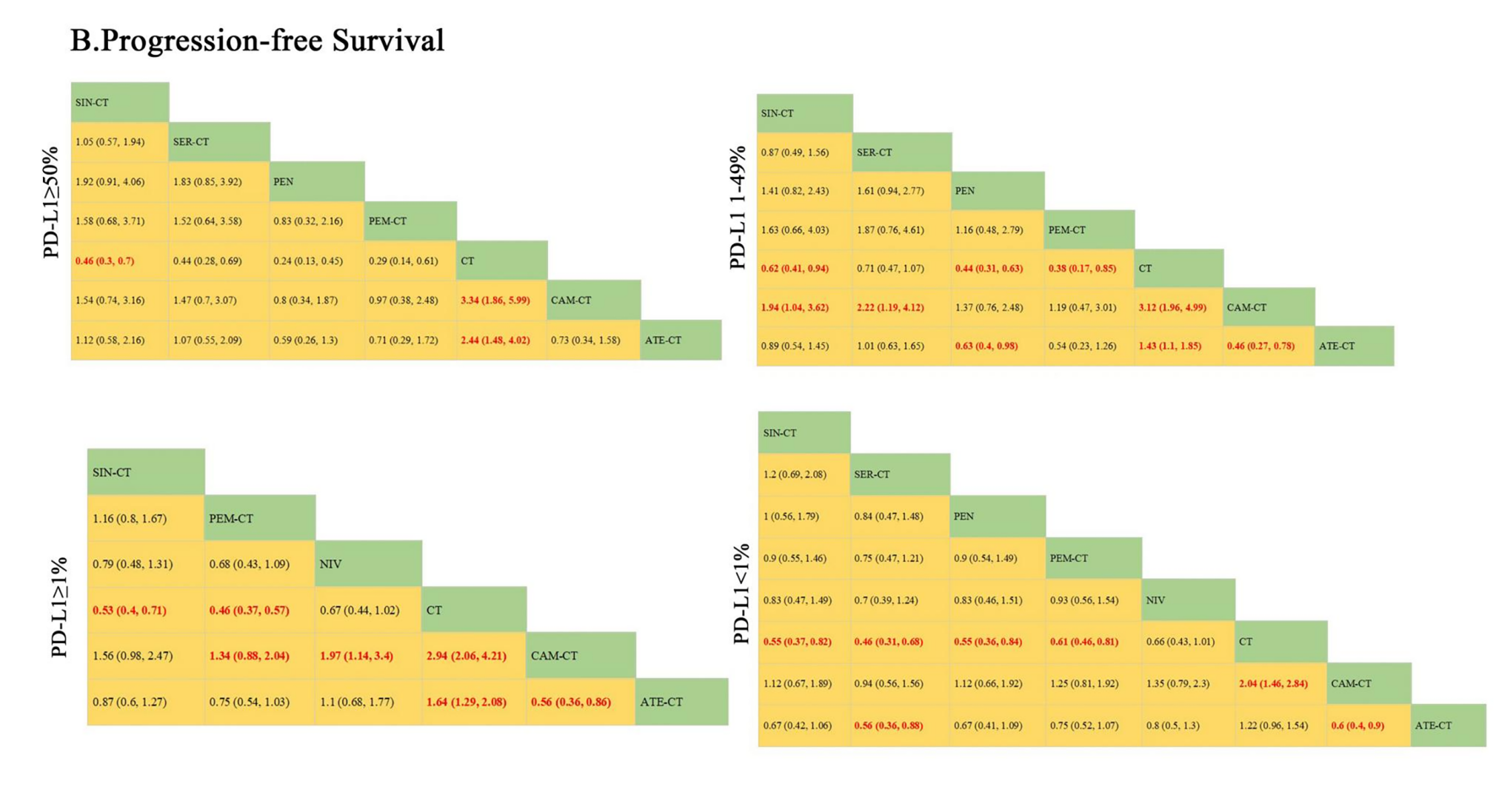


Supplementary Figure 1. Pooled estimates from a Bayesian network meta-analysis of patients with advanced squamous NSCLC based on PD-L1 expression levels (A) overall survival (B) progression-free survival.


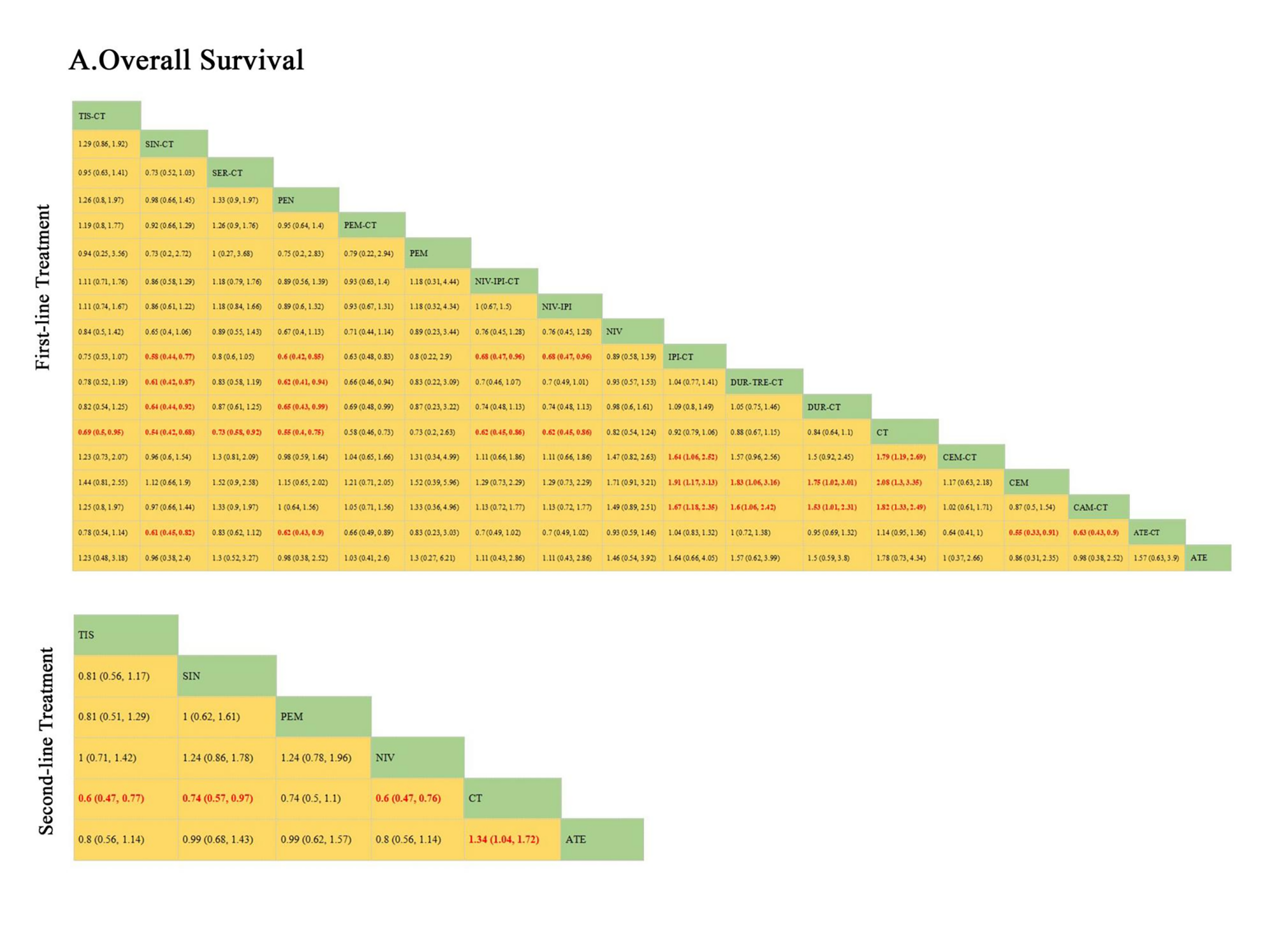


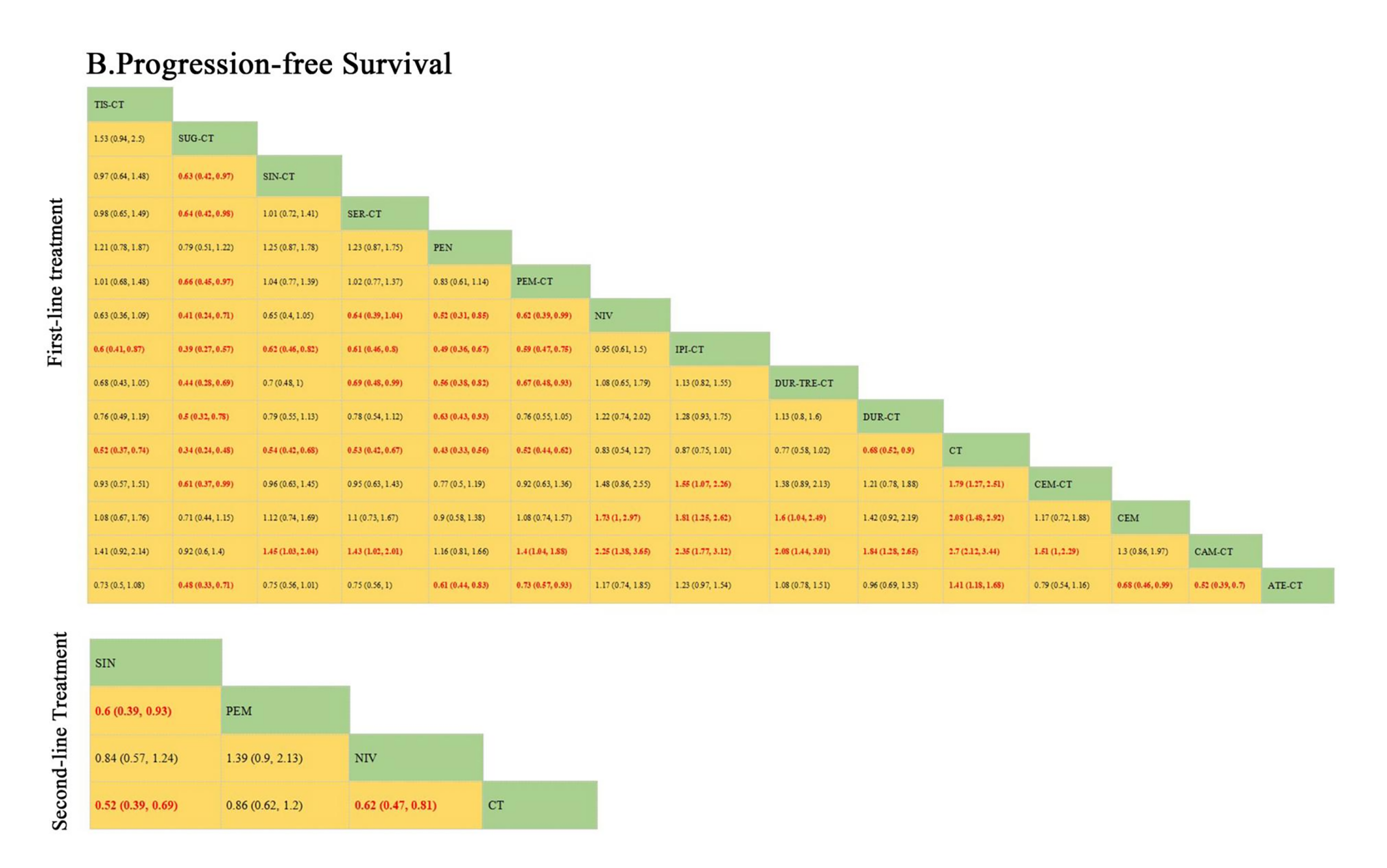


Supplementary Figure 2. Pooled estimates from a Bayesian network meta-analysis of patients with advanced squamous NSCLC according to treatment line (A) overall survival (B) progression-free survival.


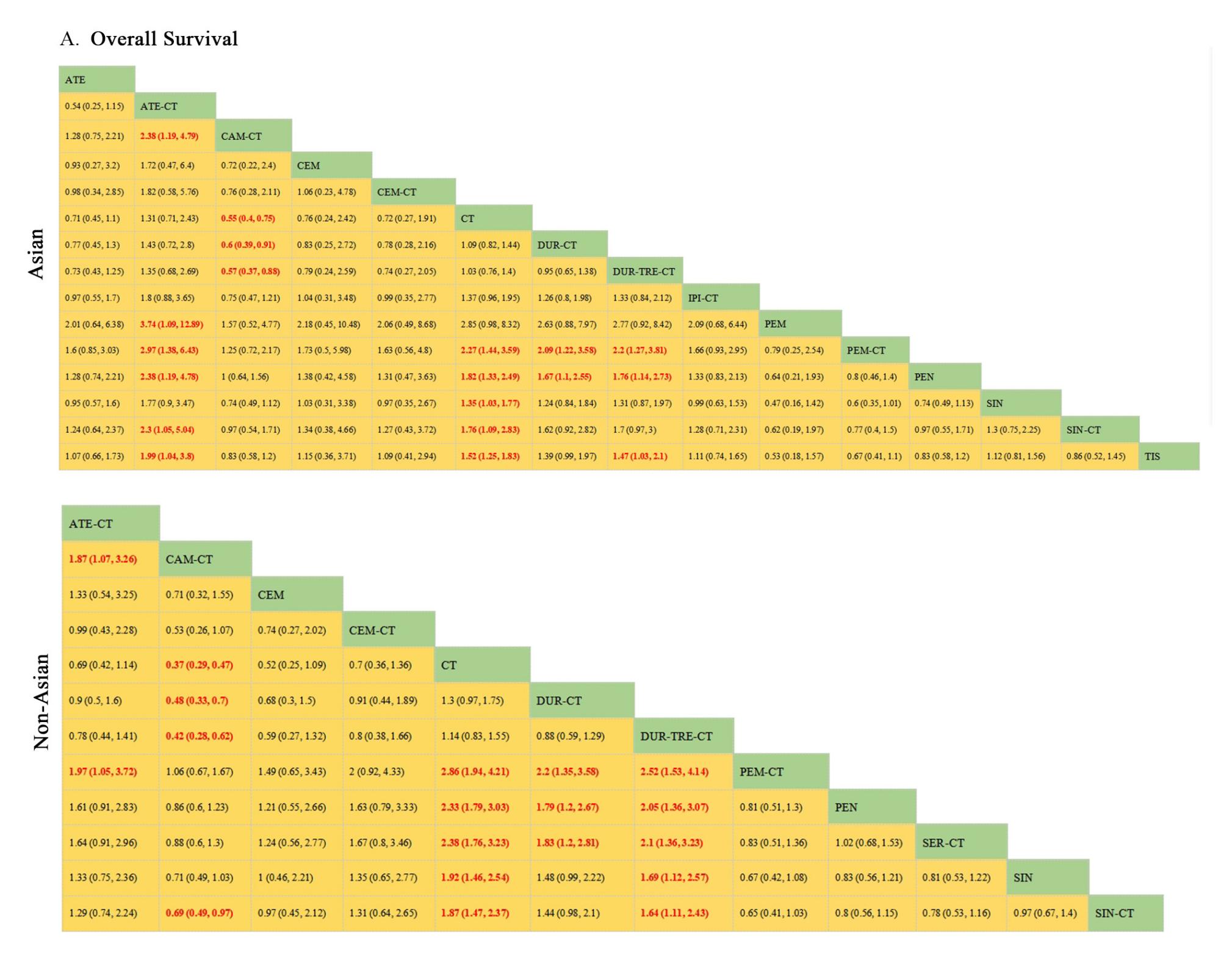


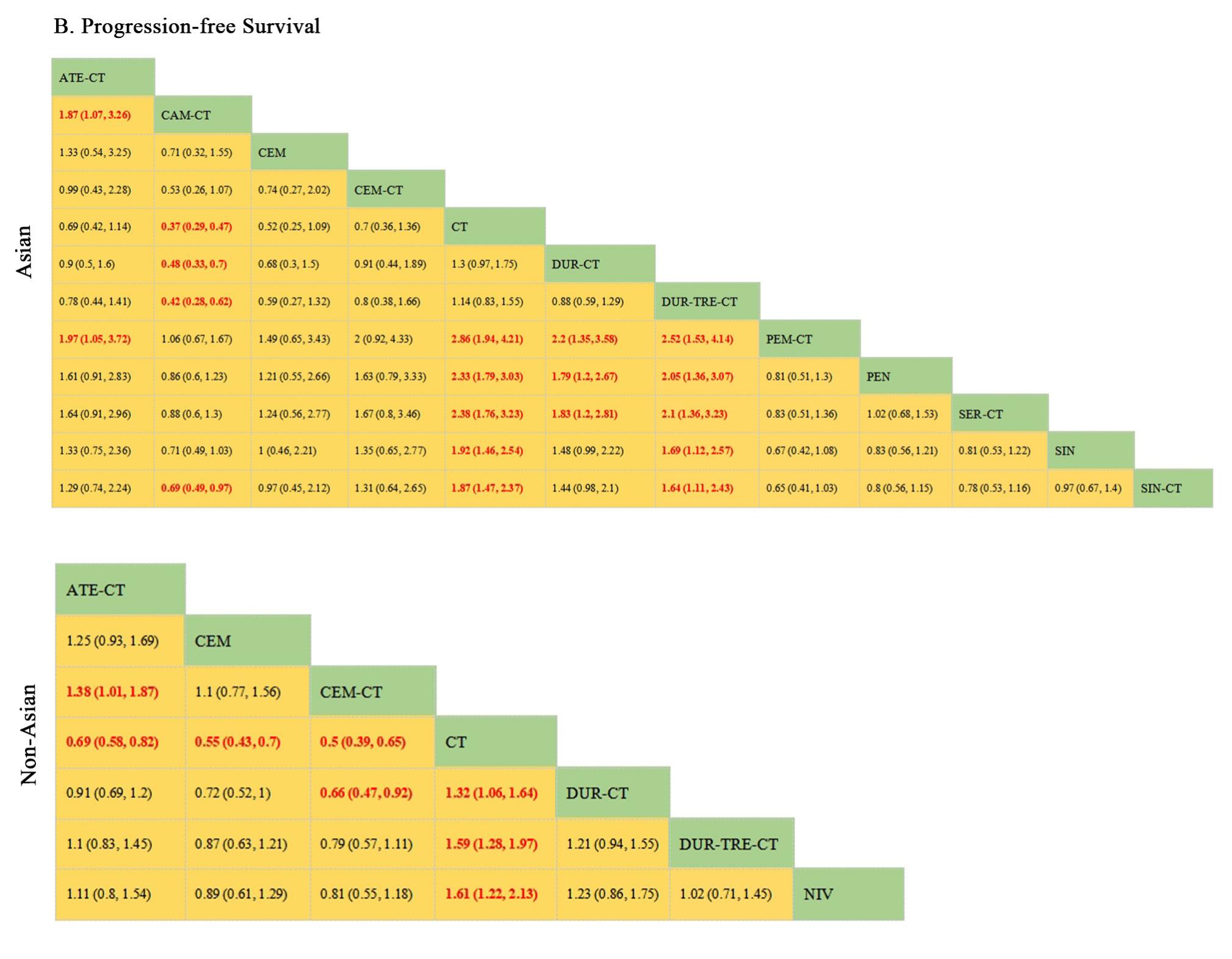


Supplementary Figure 3. Pooled estimates from a Bayesian network meta-analysis of patients with advanced squamous NSCLC according to race (A) overall survival (B) progression-free survival.


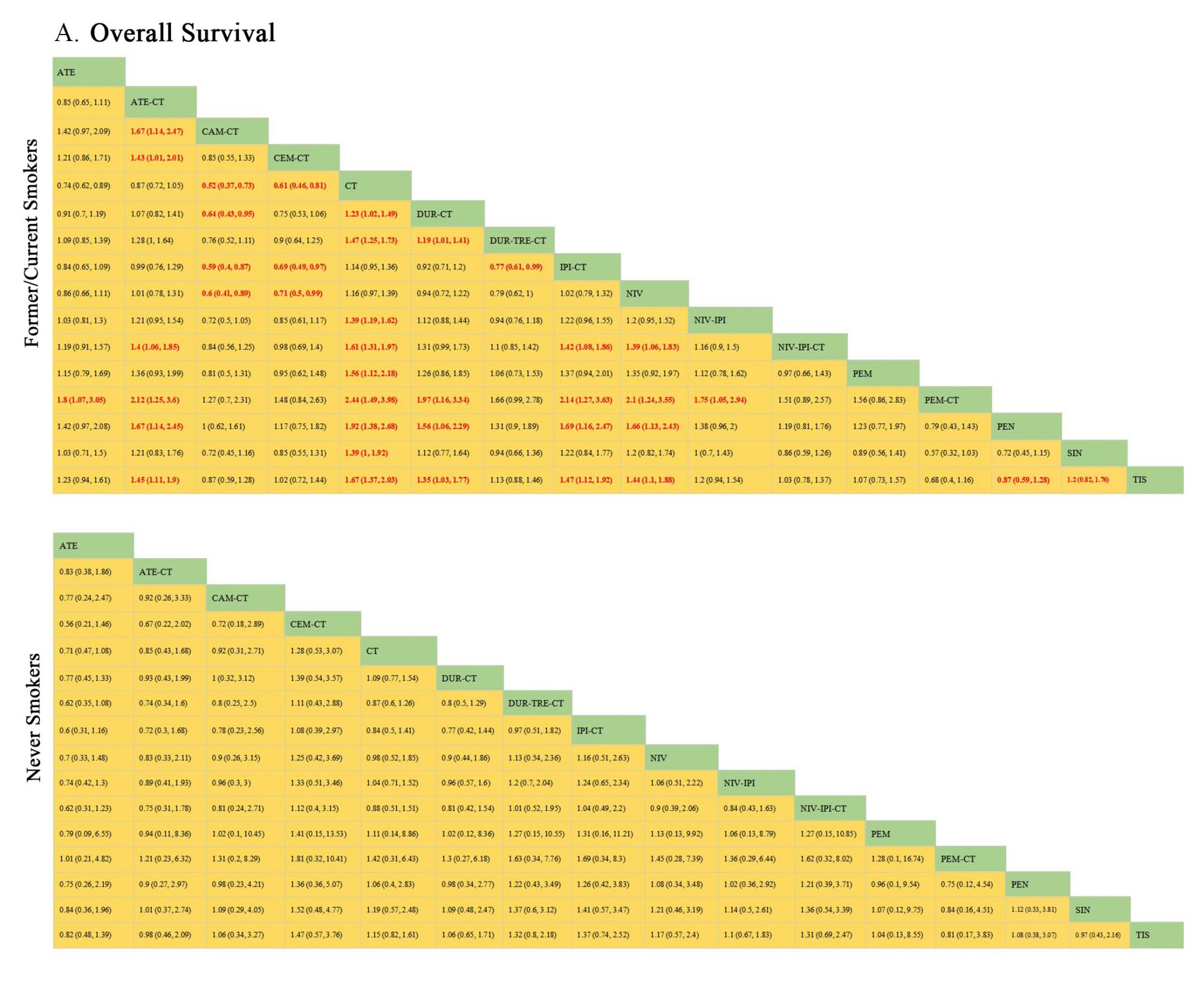


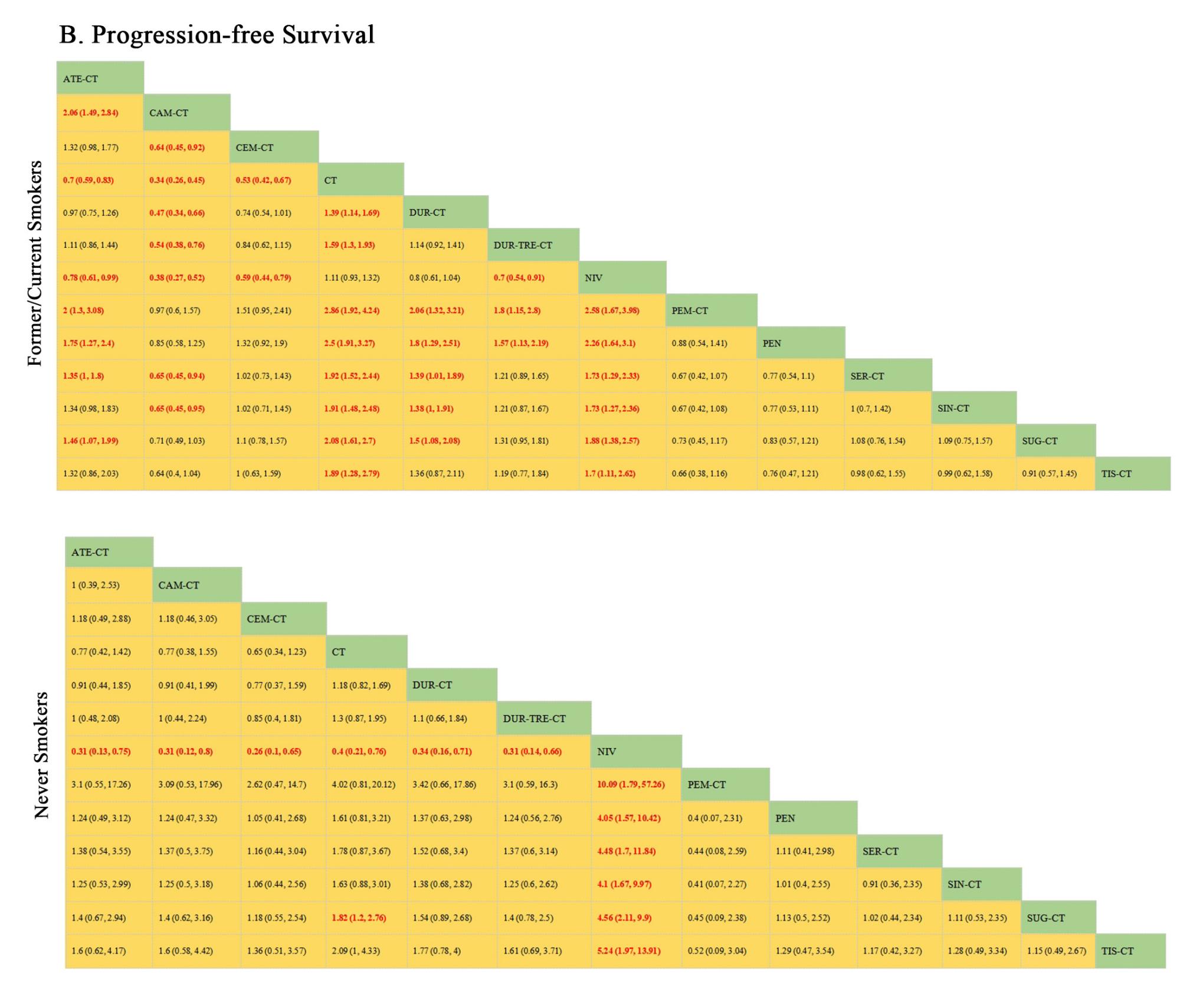


Supplementary Figure 4. Pooled estimates from Bayesian network meta-analysis of patients with advanced squamous NSCLC according to smoking history (A) overall survival (B) progression-free survival.


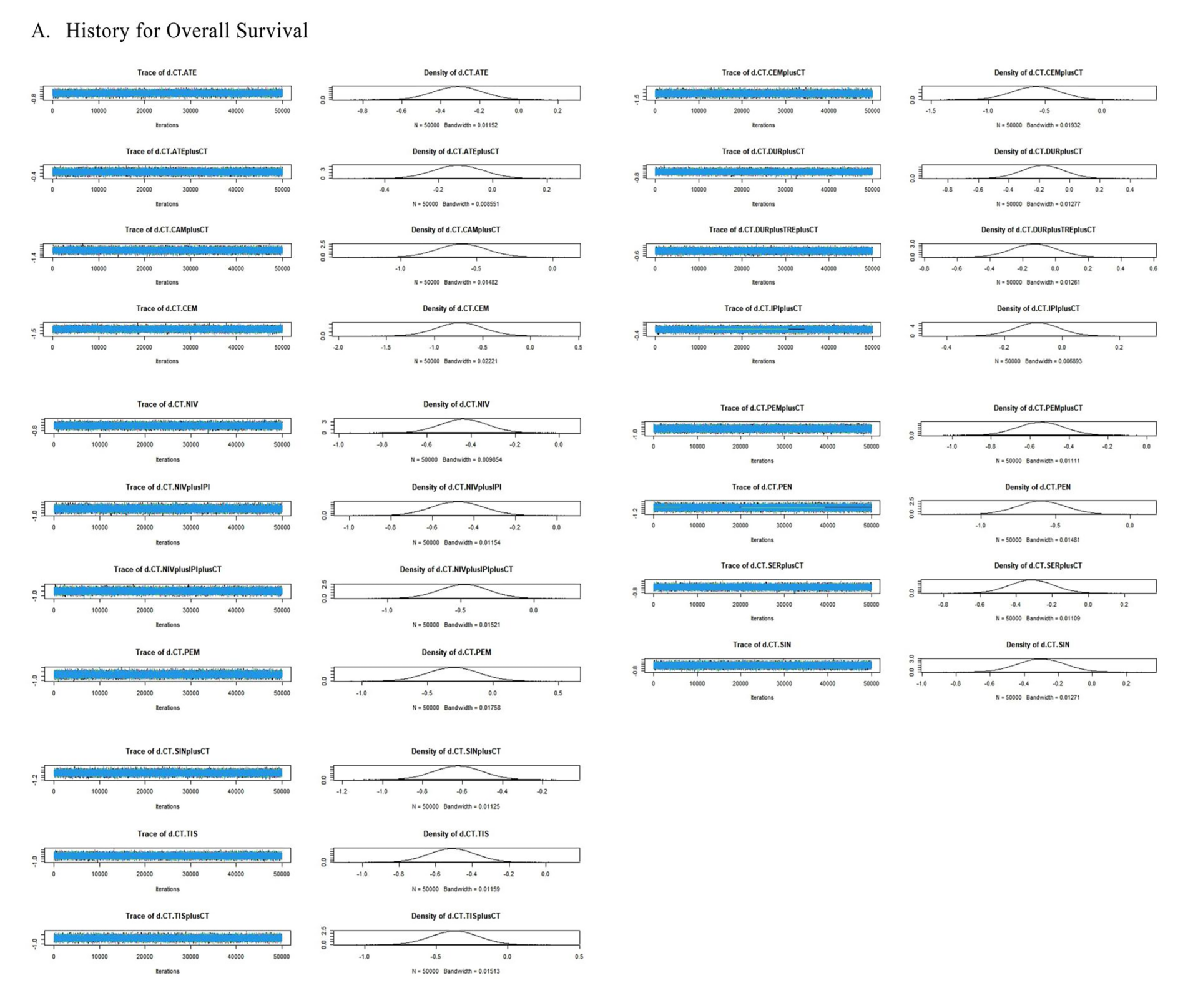


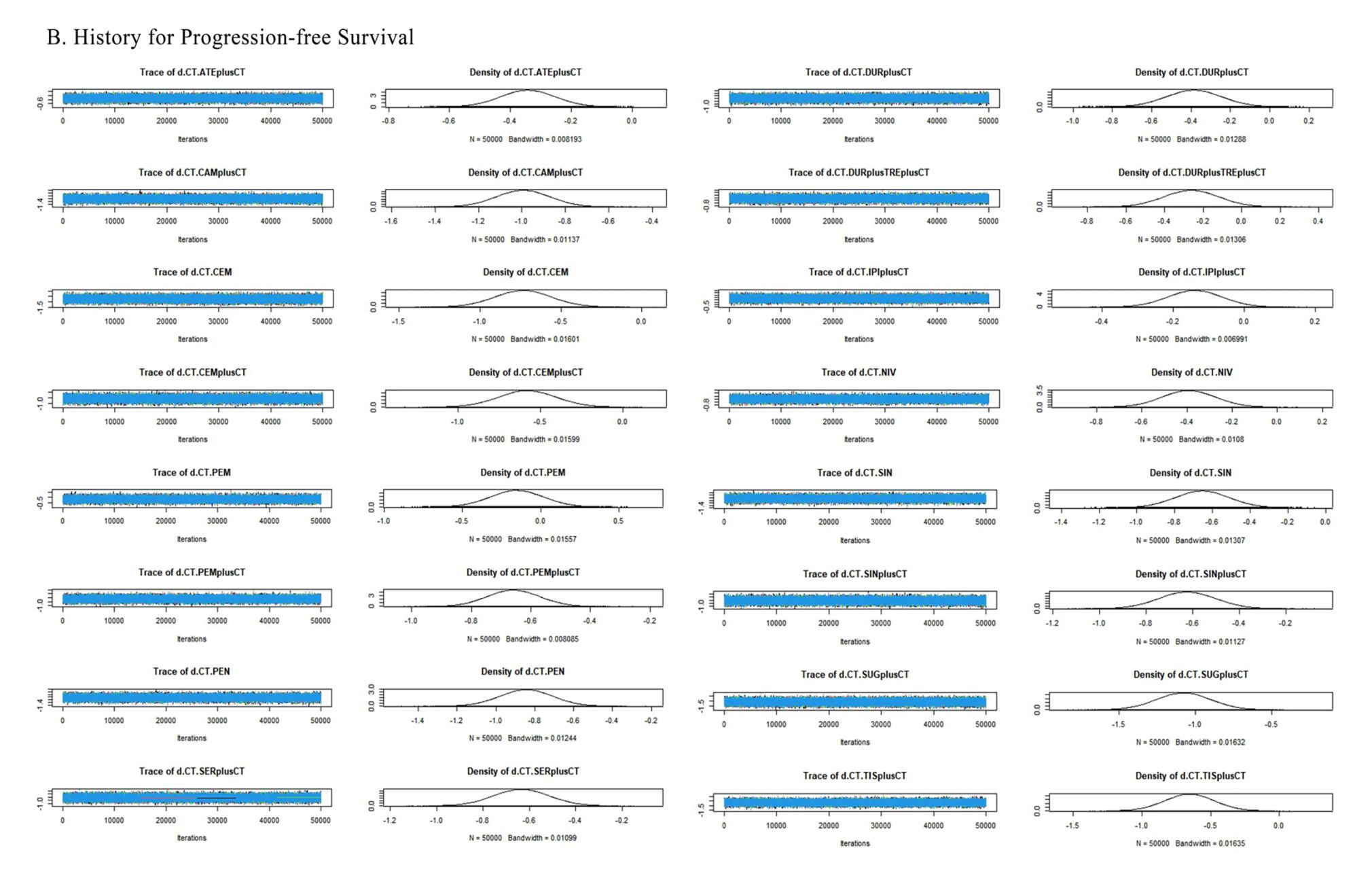


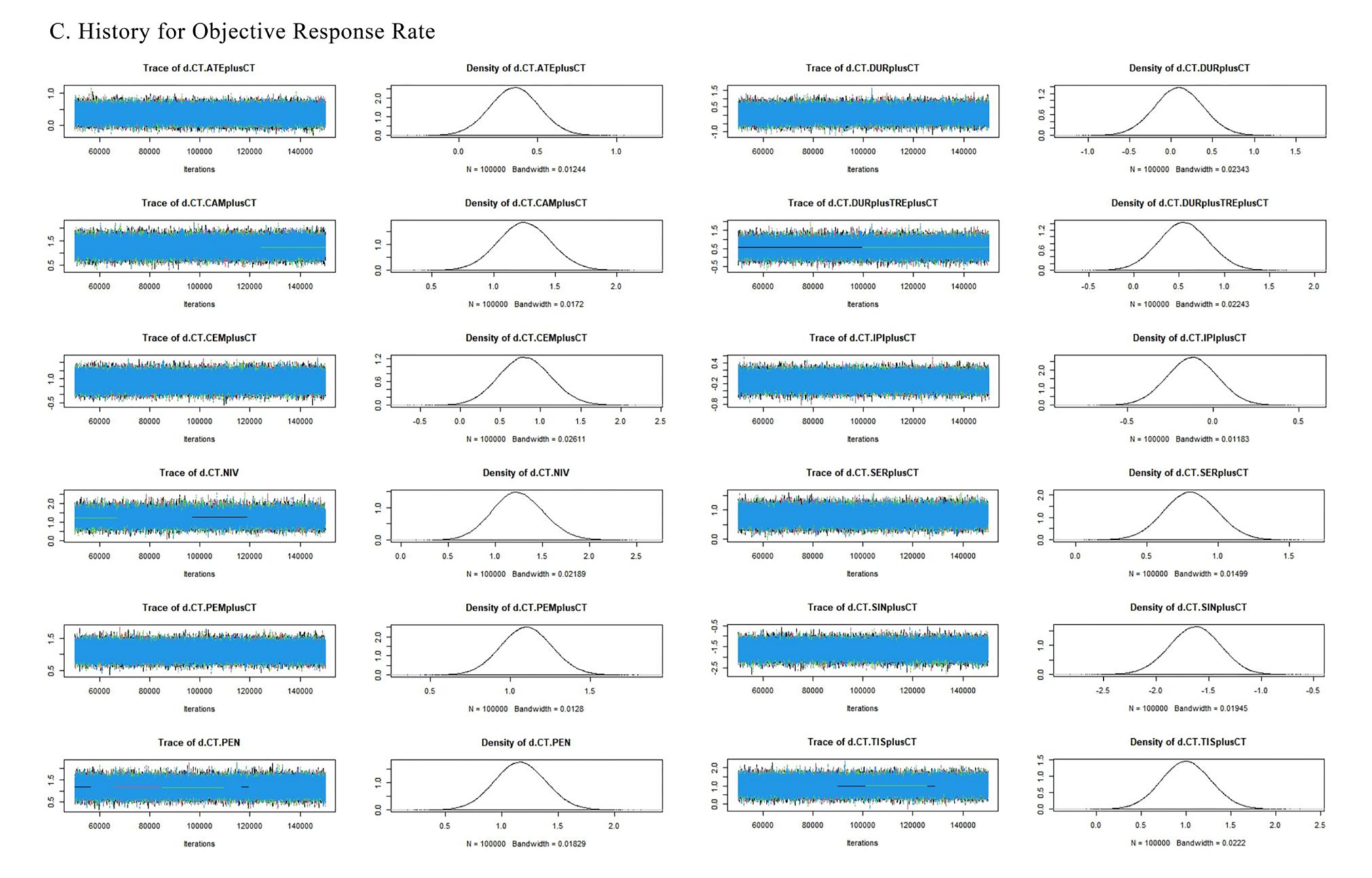


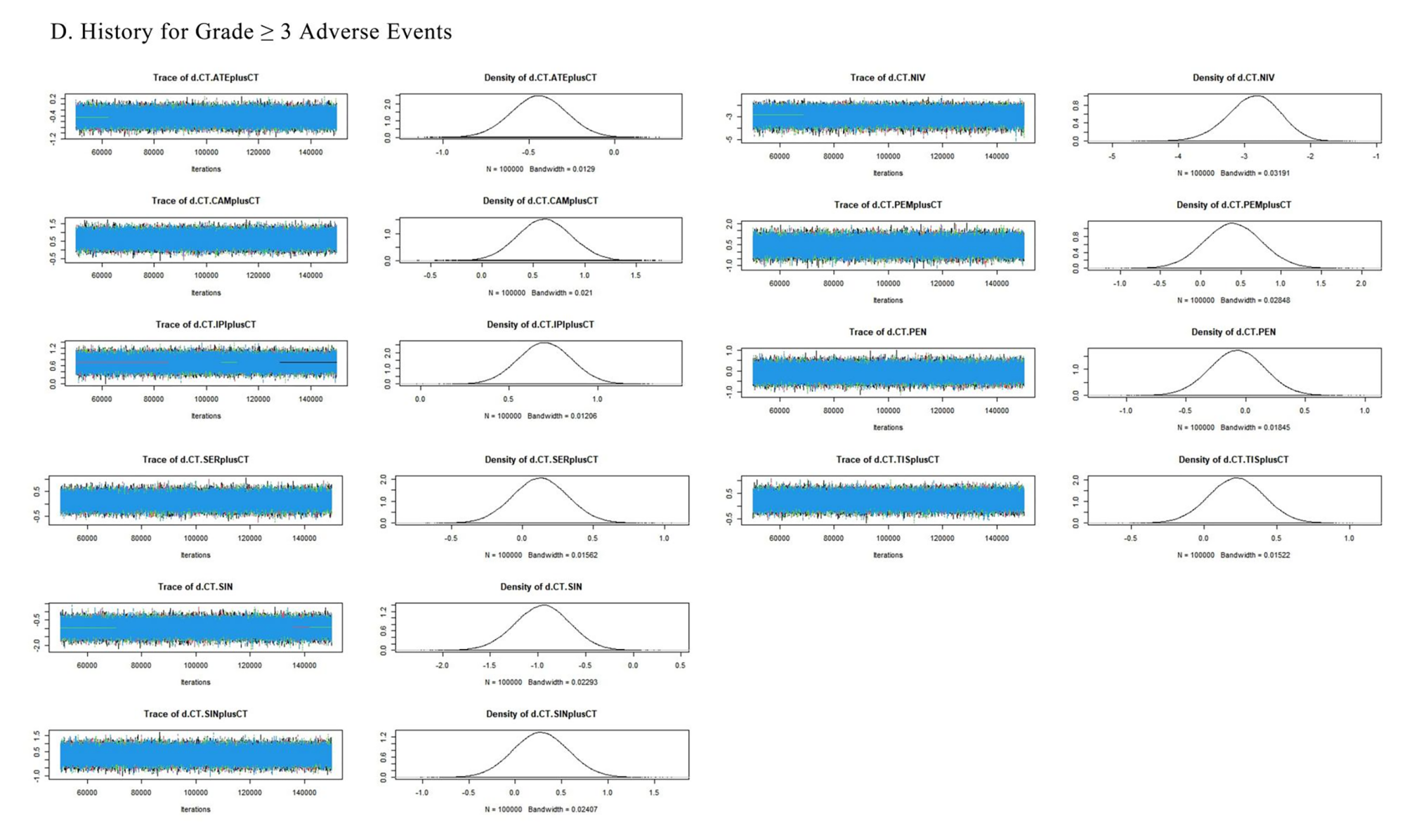


Supplementary Figure 3. convergence of three Markov chain Monte Carlo (MCMC) chains built from overall survival (A) overall survival (B) progression-free survival (C) objective response rate (D) ≥ grade 3 adverse events.


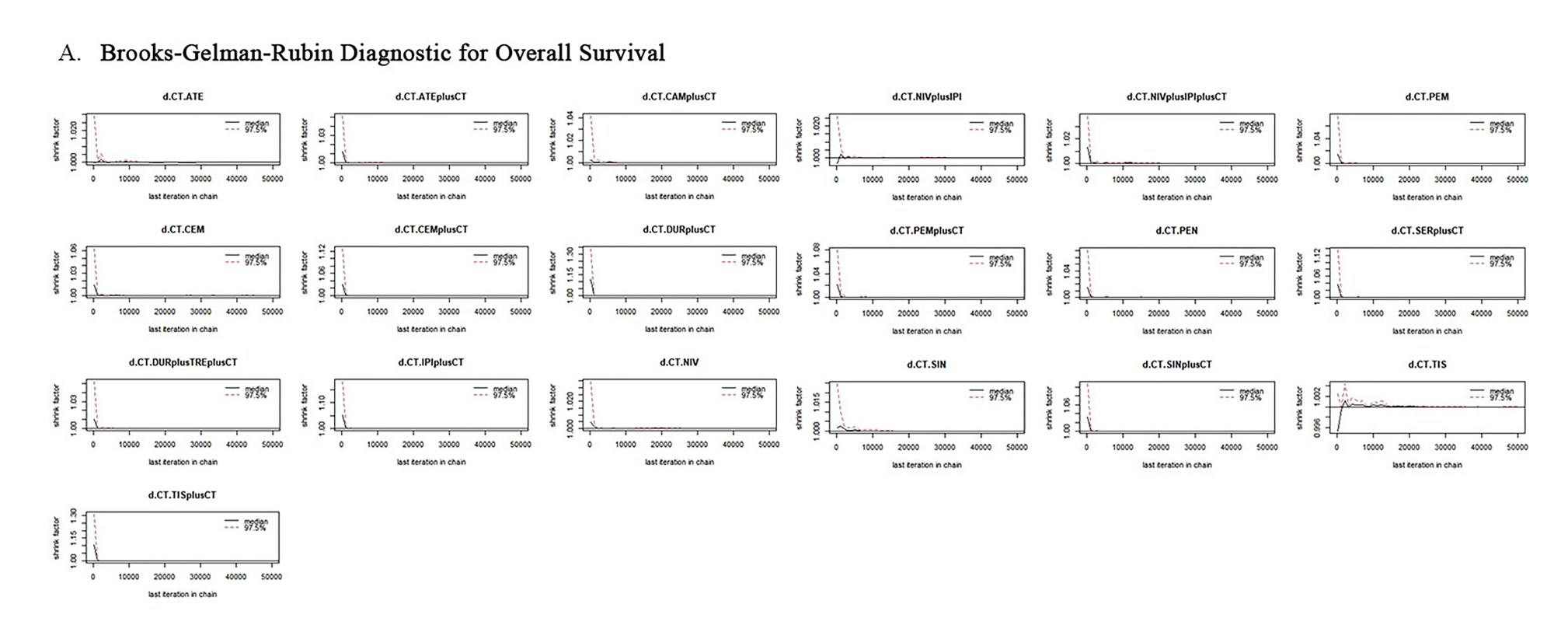


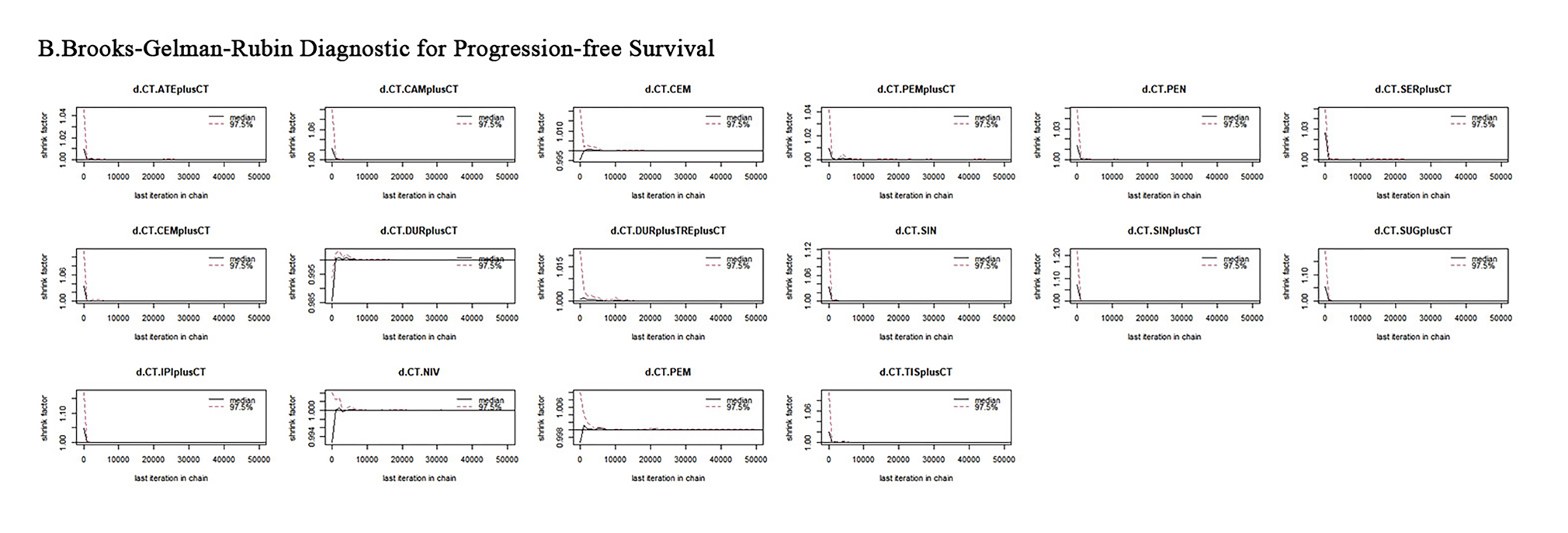


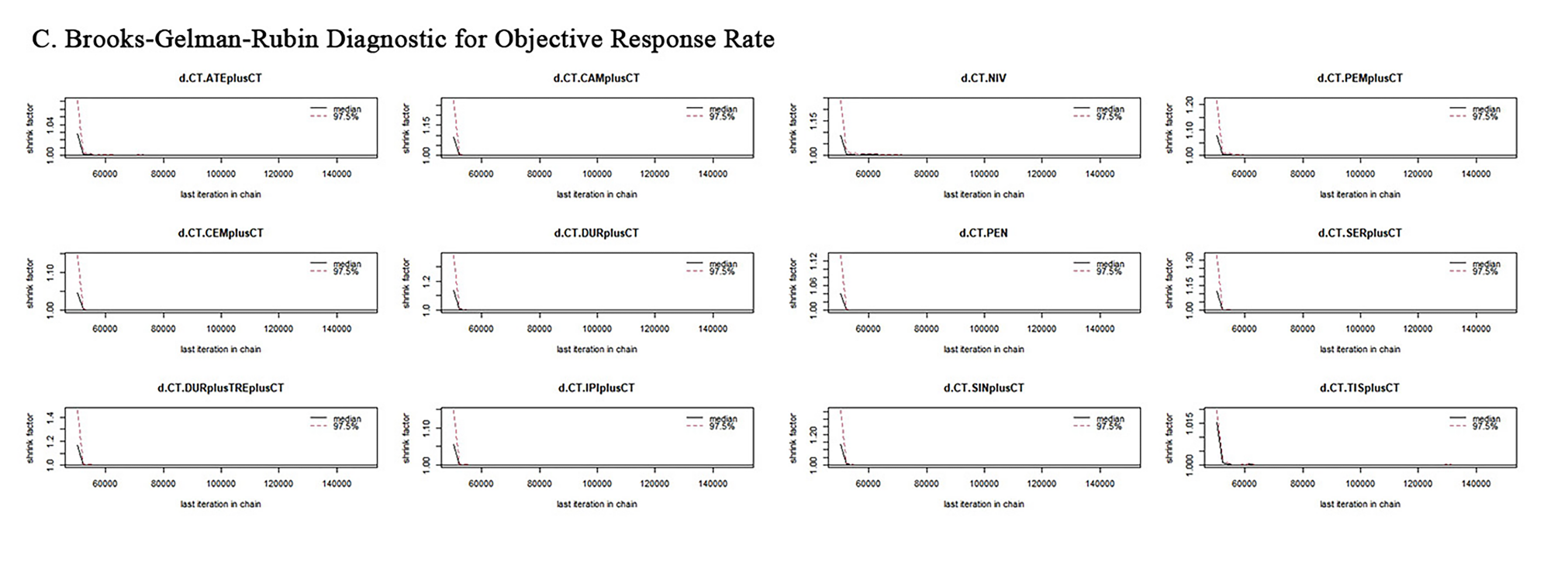


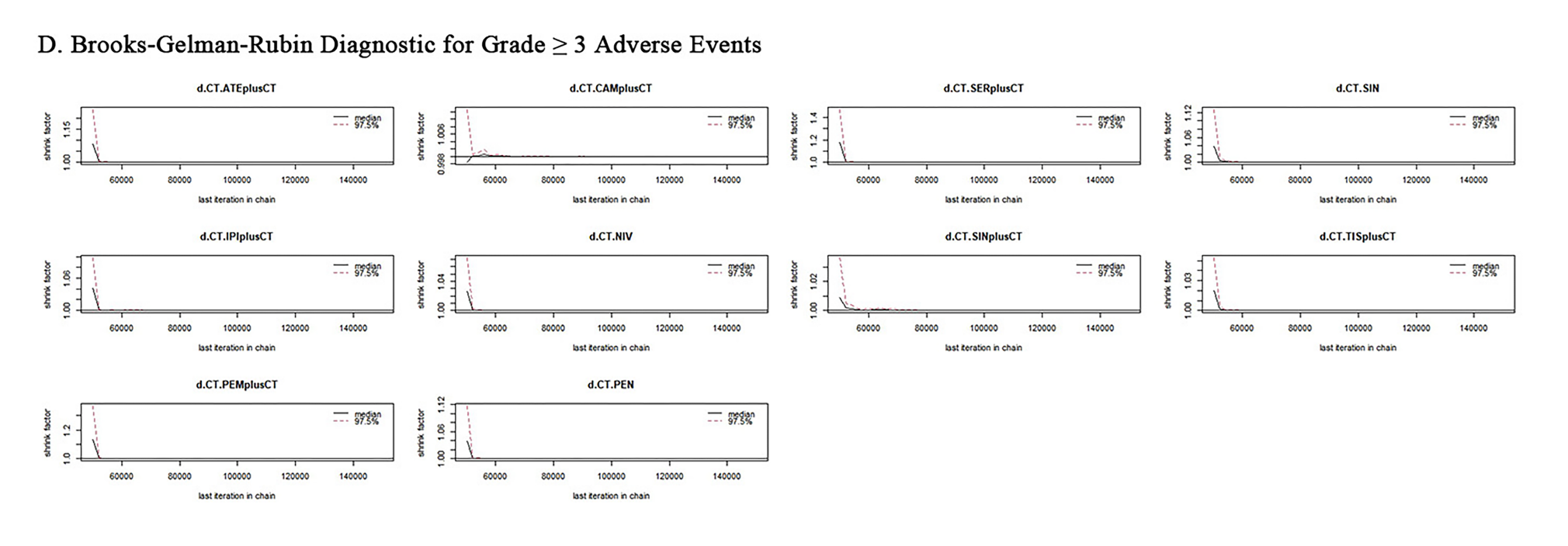


Supplementary Figure 4. Convergence of the four Markov Chain Monte Carlo (MCMC) chains established by of the Brooks-Gelman-Rubin diagnostic for overall survival (A)overall survival (B) progression-free survival (C) objective response rate (D) ≥ grade 3 adverse events.

(A)


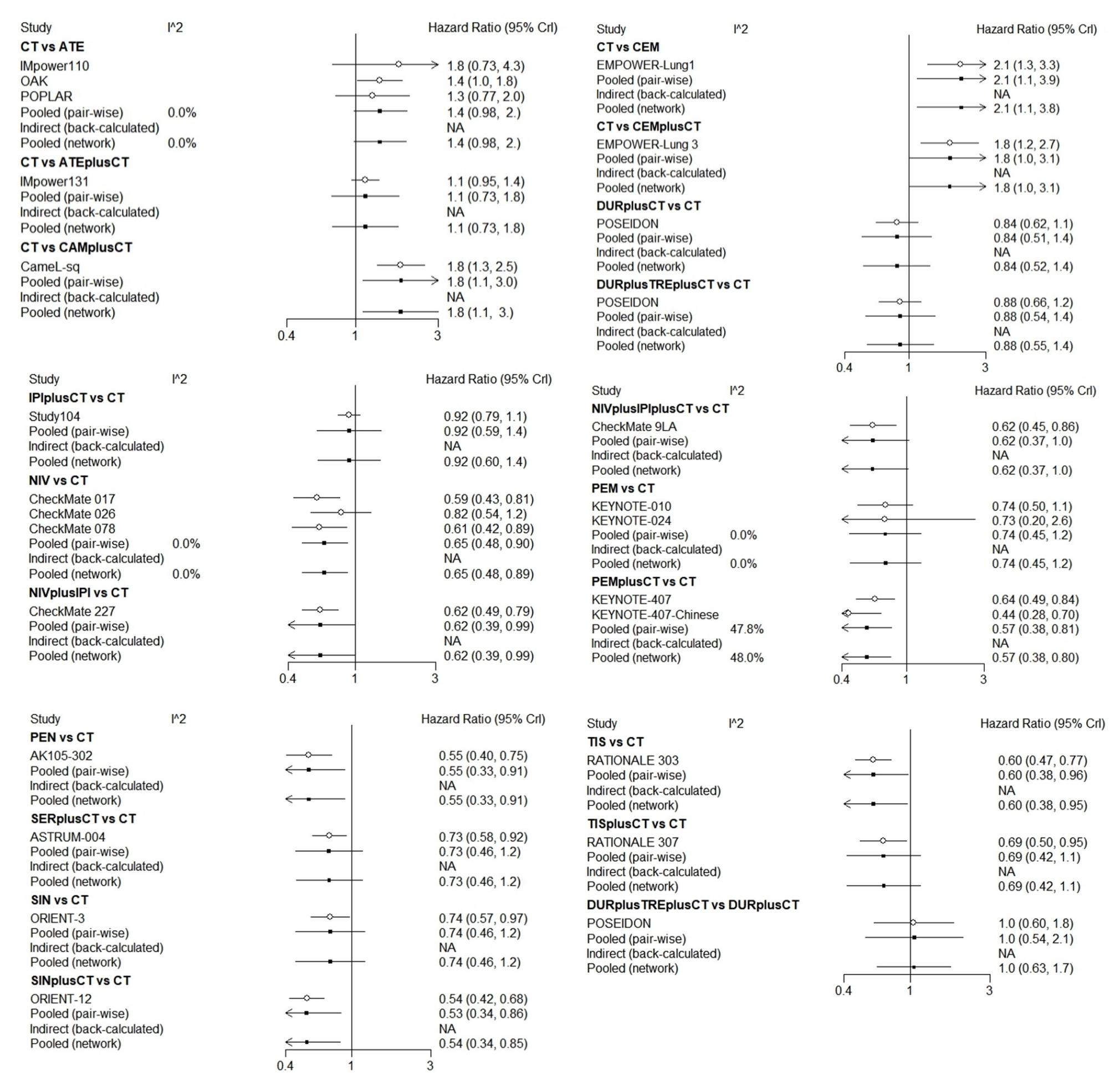


(B)


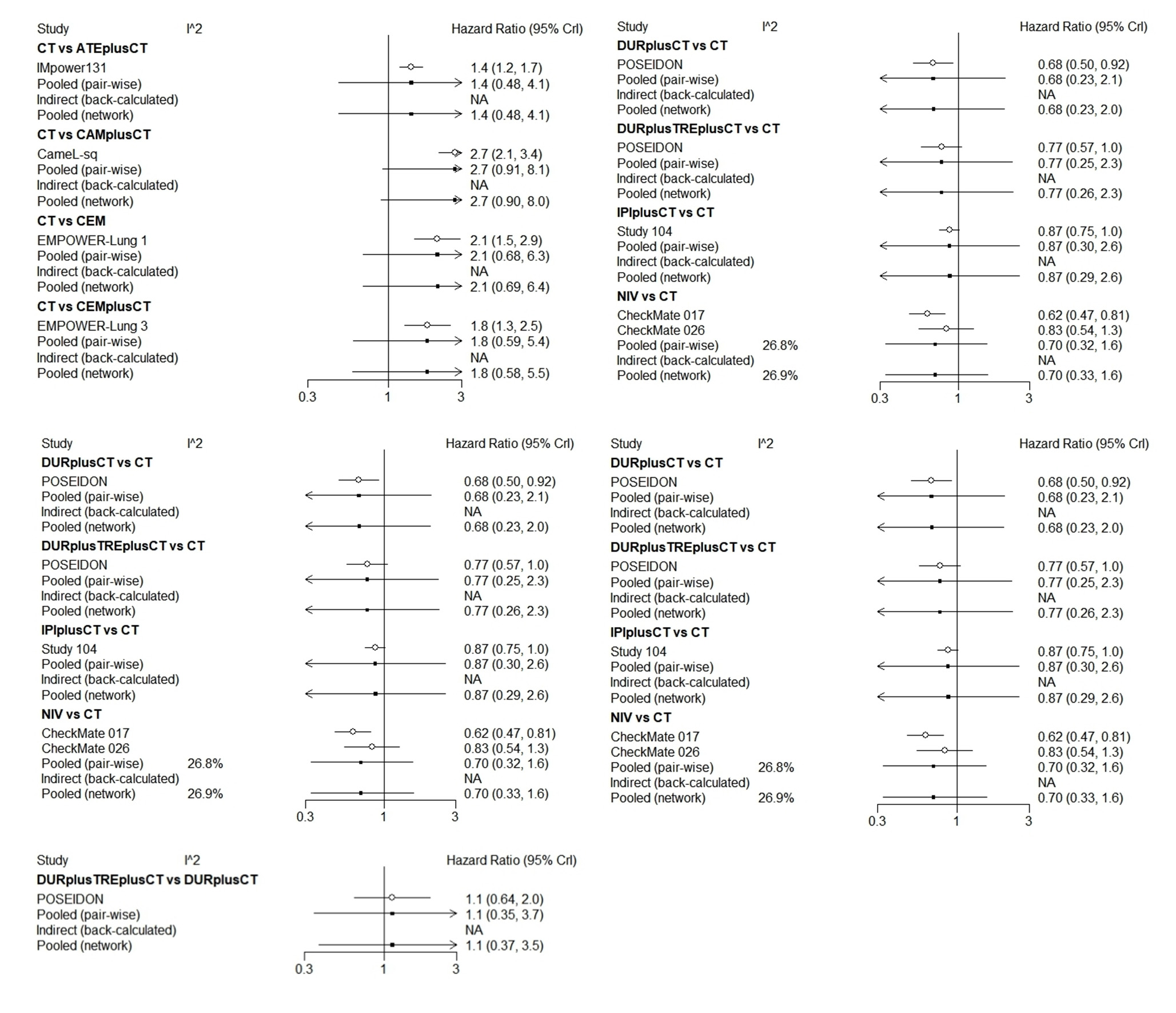


Supplementary Figure 5. Inconsistency Analysis of Network Meta-analysis Results (A)overall survival (B) progression-free survival

1. (B)


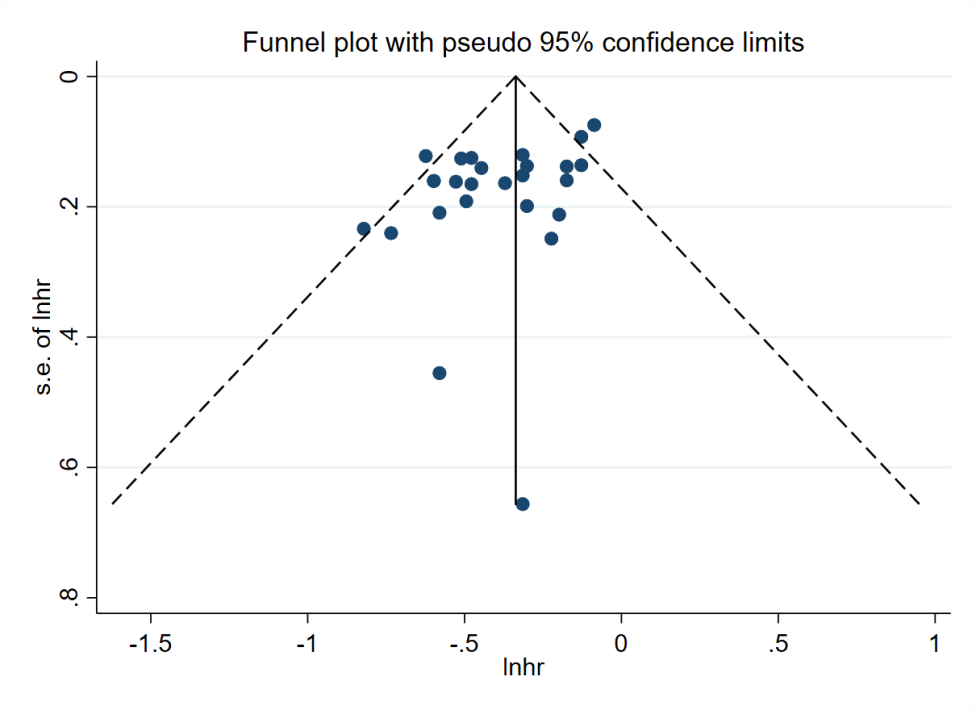

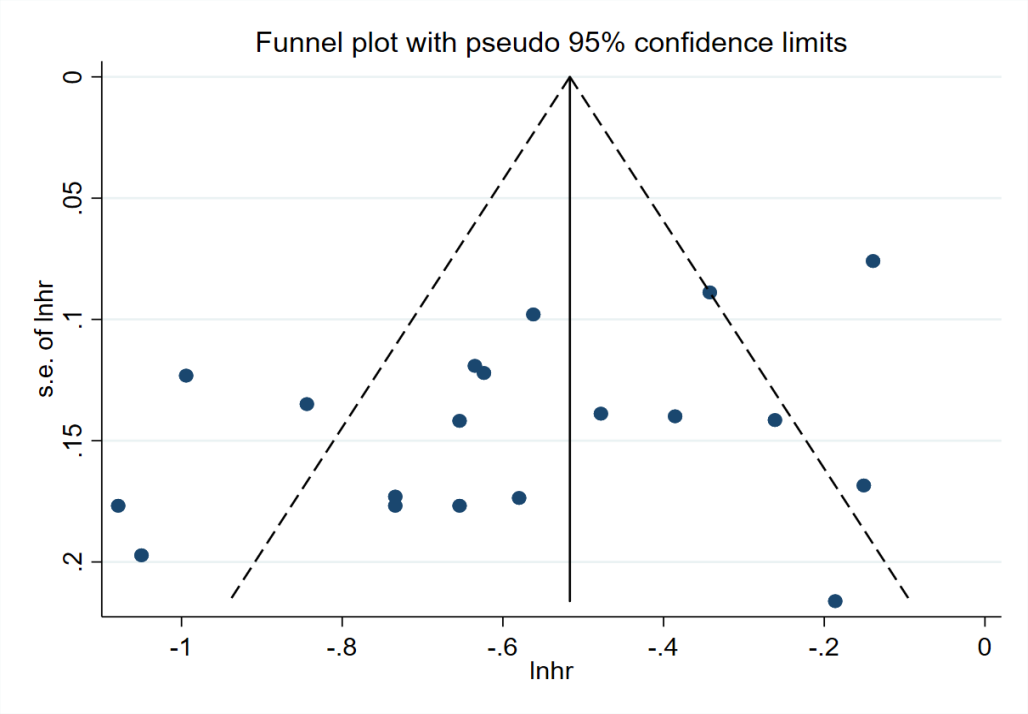


Supplementary Figure 6. Funnel plot to detect the publication bias of included studies (A)overall survival (B) progression-free survival.

Supplementary Figure 7. Egger's test for publication bias (A)overall survival (B) progression-free survival.
